# The exon junction complex is required for DMD gene splicing fidelity and myogenic differentiation

**DOI:** 10.1007/s00018-024-05188-1

**Published:** 2024-03-21

**Authors:** Dylan Da Cunha, Julie Miro, Charles Van Goethem, Cécile Notarnicola, Gérald Hugon, Gilles Carnac, Mireille Cossée, Michel Koenig, Sylvie Tuffery-Giraud

**Affiliations:** 1 PhyMedExp, Univ Montpellier, CNRS, INSERM, Montpellier, France; 2 Laboratoire de Génétique Moléculaire, CHU de Montpellier, Montpellier, France; 3 Montpellier BioInformatique Pour Le Diagnostic Clinique (MOBIDIC), Plateau de Médecine Moléculaire Et Génomique (PMMG), CHU Montpellier, 34295 Montpellier, France

**Keywords:** Duchenne muscular dystrophy, Nonsense mediated mRNA Decay (NMD), RNPS1, PININ, UPF1, Myoblasts differentiation

## Abstract

Deposition of the exon junction complex (EJC) upstream of exon-exon junctions helps maintain transcriptome integrity by preventing spurious re-splicing events in already spliced mRNAs. Here we investigate the importance of EJC for the correct splicing of the 2.2-megabase-long human DMD pre-mRNA, which encodes dystrophin, an essential protein involved in cytoskeletal organization and cell signaling. Using targeted RNA-seq, we show that knock-down of the eIF4A3 and Y14 core components of EJC in a human muscle cell line causes an accumulation of mis-splicing events clustered towards the 3′ end of the DMD transcript (Dp427m). This deregulation is conserved in the short Dp71 isoform expressed ubiquitously except in adult skeletal muscle and is rescued with wild-type eIF4A3 and Y14 proteins but not with an EJC assembly-defective mutant eIF4A3. MLN51 protein and EJC-associated ASAP/PSAP complexes independently modulate the inclusion of the regulated exons 71 and 78. Our data confirm the protective role of EJC in maintaining splicing fidelity, which in the DMD gene is necessary to preserve the function of the critical C-terminal protein–protein interaction domain of dystrophin present in all tissue-specific isoforms. Given the role of the EJC in maintaining the integrity of dystrophin, we asked whether the EJC could also be involved in the regulation of a mechanism as complex as skeletal muscle differentiation. We found that eIF4A3 knockdown impairs myogenic differentiation by blocking myotube formation. Collectively, our data provide new insights into the functional roles of EJC in human skeletal muscle.

**Supplementary Information:**

The online version contains supplementary material available at 10.1007/s00018-024-05188-1.

## Introduction

Pre-mRNA splicing that removes introns from nascent RNA during transcription elongation by RNA polymerase II, is an essential step in eukaryotic gene expression. The splicing reaction is catalyzed by the spliceosome, a macromolecular complex that assembles on splice sites at exon/intron junctions [1]. A large number of RNA-binding proteins (RBPs) cooperate to recognize authentic splice sites by interacting with multiple positive or negative cis-acting regulatory elements [2]. Exons can be spliced constitutively or alternatively in a context or developmental manner to shape tissue and cell identity [3, 4].

During splicing, the Exon Junction Complex (EJC) is loaded onto spliced mRNA in a sequence-independent manner 20–24 nucleotides upstream of exon-exon junctions as a molecular mark of splicing events [5]. The EJC consists of three core proteins, which assemble sequentially during splicing. The eukaryotic initiation factor 4A3 (eIF4A3, also known as DDX48) first binds RNA by interacting with the spliceosomal CWC22/CWC27 heterodimer [6]. Next, the pre-assembled MAGOH-Y14 heterodimer locks eIF4A3 in its RNA-bound conformation. At late stages of pre-mRNA processing, the peripheral component, metastatic lymph node 51 (MLN51) joins the trimeric core and folds around eIF4A3 ensuring EJC tight binding on the mRNA until transfer to the cytoplasm where it influences subcellular mRNA localization, translation efficiency and stability by nonsense-mediated mRNA decay (NMD) [7].

In addition to these cytoplasmic functions, there is growing evidence that EJC is also an important component and regulator of pre-mRNA processing. Data from various cellular and animal models have shown that deposition of the assembled EJC complex immediately upon splicing contributes to the production of correct mature mRNAs [8–10]. In drosophila, EJCs act positively on the splicing of long introns and suboptimal splicing substrates [11, 12] in contrast to mammalian genes, in which EJCs act rather by repressing the use of cryptic splice sites or exon-skipping events [10, 13]. An ancestral function for the conserved, sequence-independent deposition of EJC upstream of the exon-exon junction would be to protect already spliced transcripts from undesirable re-splicing events at cryptic splice sites located nearby or reconstituted at the junction after intron removal by canonical splicing [14–17]. Two peripheral complexes associated with the EJC, composed of RNPS1, SAP18 and either ACINUS (called ASAP complex) or PININ (PSAP complex), contribute to this regulation [18]. In absence or reduced expression of EJC, even splice sites with low complementarity to consensus splice motifs can be used in re-splicing events and lead to partial or complete exon loss [17]. This protective role may be crucial when introns are non sequentially removed as it facilitates re-splicing between spliced transcript segments and downstream introns that have not yet been removed [19].

The increasingly recognized importance of EJC in protecting the integrity of transcripts during splicing led us to question its role in maintaining splicing fidelity in very large human mRNAs such as those encoding skeletal muscle-specific structural proteins. An important gene is the Duchenne Muscular Dystrophy (DMD) gene, the largest in the human genome (2.2 Mb), which comprises 79 exons scattered among huge introns comprising multiple competing cryptic splice sites that can alter the integrity of the mature transcript produced. It encodes dystrophin, an essential cytoskeletal protein of 427 kDa that is defective in Duchenne Muscular Dystrophy, the most common and severe muscular dystrophy in children [20]. Dystrophin links the internal cytoskeleton to the extracellular matrix as part of the dystrophin-glycoprotein complex (DGC). In addition to the skeletal muscle isoform (Dp427m), two full-length isoforms (Dp427b, Dp427p) and four shorter isoforms (Dp260, Dp140, Dp116, Dp71) isoforms are expressed from alternative tissue-specific promoters [21]. Using a DMD-targeted RNA-seq approach, we have previously established that, unlike other isoforms expressed in the brain such as Dp71 [22], the Dp427m transcript is not alternatively spliced [23]. All the 79 exons are included in mature transcripts to produce a full-length dystrophin protein in skeletal muscle. We identified some of the RBPs that contribute to exon inclusion in this isoform [24]. However, the molecular basis of splicing regulation in the DMD gene remains largely unexplored.

Here, we provide new insights into the mechanisms that help preserving splicing fidelity in the large DMD pre-mRNA. By profiling splicing changes induced by the knockdown (KD) of EJC core components and peripheral ASAP/PSAP complexes in a human muscle cell line, we show that normal expression of EJC and associated proteins is necessary to maintain the integrity of the 3’end of the DMD transcript. This region, encoding the critical C-terminal protein domain, is involved in anchoring dystrophin in the sarcolemma and in interactions with signaling proteins. In addition to its impact on DMD splicing, our data reveal that inhibition of eIF4A3 expression also impairs myoblast differentiation, and that the NMD factor UPF1 appears to contribute to normal dystrophin expression.

## Materials and methods

### Cell culture

The human immortalized myoblast cell line C25Cl48 (KM155C25), established from a muscle biopsy of a 25-year-old healthy individual [25] can differentiate by fusing into long multinucleated myotubes that express specific skeletal muscle proteins. These cells were cultured in a specific proliferation medium as previously described [26]. Myogenic differentiation was induced for 3 days by serum deprivation and elevation of insulin concentration to 10 µg/mL in the medium. At this stage, myotubes express high levels of dystophin mRNA and proteins [24]. HeLa cells were grown in Dulbecco’s modified Eagle’s medium/ F-12 (DMEM/F-12, Invitrogen) supplemented with 10% (v/v) fetal bovine serum (Eurobio), 1% of l-glutamine and 1% of non-essential amino-acids (ThermoFisher Scientific).

### siRNA transfection procedures

For *DMD*-targeted RNA-seq experiments, transfection of siRNAs was performed as previously described [24]. Briefly, C25Cl48 cells were reverse transfected with control (siGENOME non-targeting siRNA#5), eIF4A3 or Y14 siRNA (Dharmacon) at a final concentration of 37.5 nM in 10 cm cell culture dishes using 44.25 µL Lipofectamine RNAiMAX (ThermoFisher Scientific) following the manufacturer’s instructions. After 48 h, a second round of siRNA transfection was performed and the cells were incubated in differentiation medium for 3 days. In other KD experiments, C25Cl48 cells were seeded into 6-well plates (1.5 × 10^5^ cells/well) and a single round of reverse transfection was carried out with 37.5 nM siRNAs targeting EJC, ASAP/PSAP or NMD components. The source and target sequences of the siRNAs used are listed in the Supplementary Table 1. Cells were harvested in Trizol for RNA analysis and in Laemmli sample buffer supplemented with 5% of β-mercaptoethanol or RIPA buffer (50 mM Tris–HCl [pH 7.4], 150 mM NaCl, 1% NP-40, 0.1% SDS, 0.5% Na-deoxydiolate) when specified, for protein analysis. Knockdown efficiency was assessed by RT-qPCR and western blot analysis. Experiments were performed in four independent biological replicates.

### DMD-targeted RNA-seq experiments and computational analysis

*DMD*-targeted RNA-seq was performed as previously described [23, 24] on two (eIF4A3 and Y14 siRNA) and four (control siRNA) independent biological replicates. The full-length DMD cDNA produced by priming with oligo(dT) (Superscript II, Thermofisher scientific) was amplified as a single long fragment (11.3 kb) by Long-Range PCR (LR-PCR) (GoTaq^®^ Long PCR Master Mix, Promega) using primers located in exon 1 and the 3′ UTR of the muscle isoform (Dp427m) (Supplementary Table 1). The libraries were prepared from 1.6 ug of purified LR-PCR products using the Rapid Library Preparation Kit (Roche) and multiplexed libraries were sequenced at a minimum read depth of 346X on the Roche GS Junior 454 sequencer. The sequencing data were analyzed as previously described [24]. After adapter and quality-based trimming, cleaned reads of mean length of 322 bp were aligned against the GRCh37/hg19 human X chromosome reference sequence using the STAR read aligner (v2.3). The summary of mapping statistics is provided in the Supplementary Table 2. The number of split reads supporting exon inclusion and exclusion was used for calculation of exon centric and intron centric splicing metrics with the Integrative Pipeline for Splicing Analyses (IPSA) software [27]. The exon inclusion rate, known as Percent-Spliced-In ratio (PSI), was computed for all annotated 79 exons of the Dp427 m isoform and the average PSI values from replicates were used to compare experimental conditions (control, eIF4A3 KD, Y14 KD). In this calculation, only reads that span to the adjacent exons are considered, revealing alternative splicing events that correspond to the defined single exon skipping events. To allow detection and quantification of any other novel alternative splicing event (ie multiple exon skipping events, 5′- or 3′-alternative splice site, inclusion of cryptic exon) the IPSA intron-centric metrics, which independently measures splicing at the 5′ and 3′ end of the intron (psi5 and psi3 indices, respectively), was employed. The differential usage of a novel splice junctions (SJ) supported by at least 5 reads in both eIF4A3 KD or Y14 KD replicates, was calculated as follows: ∆SJ = SJ(KD)–SJ(Ctrl). Alternative splicing events with greater than 5% variation are reported in the Supplementary Table 2. To visualize splicing events, sashimi plots were generated with ggsashimi [28], reporting the mean read counts (from the two biological replicates) across splice junctions obtained using the alignment files, in BAM format, produced by STAR.

### Reverse transcription and real-time quantitative PCR (qPCR)

Total RNA was isolated from cells using Direct-zol RNA MiniPrep (Ozyme) according to the manufacturer’s instructions. Reverse transcription (RT) was performed on 300 ng total RNA using random hexamer primers and SuperScript II reverse transcriptase (Life Technologies) following the manufacturer’s conditions. For quantitative PCR (qPCR), 1 µL of 1:6 diluted cDNA was amplified in triplicate technical repeats of each PCR condition using the SYBR Green I Master Mix and LightCycler 480 (Roche) in a final volume of 5 µL containing 0.5 µM of primers targeting the cDNA of interest. Amplified transcripts were quantified using the comparative CT method normalized to RPLP0 expression (Hs_RPLP0_2_SG QuantiTect Primer Assay, Qiagen). The results are presented as mean ± SD of normalized fold-change expression (2^–ΔΔCT^) for assessment of siRNA efficiency or expression levels (2^–ΔCT^) for the analysis of dystrophin and differentiation factors expression.

### End-point PCR and semi-quantitative fluorescent PCR (QFPCR)

All PCR experiments involved four independent biological replicates. For conventional end-point PCR, primers in exons flanking the alternative splicing event were used to amplify 1 μL of cDNA with the Taq DNA polymerase (New England Biolabs) for 35 cycles in a 25 μL total volume. RT-PCR products were resolved by gel electrophoresis in 2% agarose gels and bands were quantified using the Image Lab 6.0.1 software (Bio-Rad). The identity of the amplified products was verified by Sanger sequencing. For semi-quantitative analysis of fluorescently marked PCR products, the same protocol was applied except that FAM-labeled forward primers were used and the cycling conditions were as follows: 94 °C for 3 min followed by 26 cycles at 94 °C for 30 s, 59 °C for 30 s and 72 °C for 45 s and a final extension step at 72 °C for 20 min. Fluorescently labeled PCR fragments were separated by size on an Applied Biosystems^®^ 3500 Dx Genetic Analyzer and analyzed with the GeneMapper v6.0 software (Applied Biosystems). Ratios of splicing isoforms were determined as the peak area of one specific isoform divided by the total peak areas for the other detected isoforms. Primer sequences for RT-PCR are provided in the Supplementary Table 1.

### siRNA rescue experiments

HeLa cells were reverse transfected in 6-well plates (1.8 × 10^5^ cells/well) with control, eIF4A3 or Y14 siRNAs at a final concentration of 37.5 nM and 7.5 μl Lipofectamine RNAiMAX (Thermo Fisher Scientific). Cells were transfected 24 h later with 1 μg of plasmids expressing FLAG-tagged proteins or empty vector (negative control) using 6 μl Lipofectamine^®^ LTX (Life Technologies) according to the manufacturer’s protocol. Cells were harvested 24 h post-transfection of the plasmids. Expression of the tagged protein was verified by western blot analysis and purified RNA was used for reverse transcription. The siRNA-resistant plasmids for the wild-type eIF4A3 protein (FLAG-eIF4A3-WT) and a mutant form (D401KE402R) that does not form EJC (FLAG-eIF4A3-Mut) [10] are kind gifts from Dr H. Le Hir (IBENS, Paris, France). We have constructed the siRNA-resistant plasmid for the wild-type Y14 protein (FLAG-Y14-WT) by cloning the amplified coding region of Y14 into the NheI/XhoI sites of a pcDNA3 plasmid. To make the construct siRNA-resistant, four silent point mutations were introduced in the siRNA target region at the third position of codons using the Quick-Change site-directed mutagenesis kit (Agilent). The primers used are described in the Supplementary Table 1 and all constructs were verified by Sanger sequencing.

### Western blot analysis

After ultra-sonication of cell lysates, equal amounts of total proteins were separated by electrophoresis on 10% or 4–15% precast (for the analysis of Y14 and dystrophin proteins) SDS-Polyacrylamide gels (Thermo Fisher Scientific). Proteins were transferred onto nitrocellulose membranes using Trans-Blot Turbo Transfer System (Biorad) and probed with specific antibodies against eIF4A3, Y14, MLN51, RNPS1, SAP18, ACINUS, PININ, UPF1, dystrophin, Myosin (skeletal, Fast) Heavy Chain (MyHC) and Troponin T. Anti-ß-Tubulin or GAPDH was used as loading controls. The origin of the antibodies and dilutions used are described in the Supplementary Table 1. After washing, membranes were probed with secondary antibodies either conjugated to horseradish peroxidase (HRP) or labeled with LI-COR IRDye fluorescent dye (Thermofisher). Protein signals were detected on an Azure Saphirre™ imager (Azure Biosystems) using Enhanced chemiluminescence ECL reagents (Pierce) or visualized by an Odyssey infrared imaging system (Li-COR Biosciences). Western Blotting images were collected and stacked on ImageJ software (v2.0.0) and the densitometry was performed using Image Lab 6.0.1 software (Bio-Rad).

### Immunofluorescence microscopy and myotubes analysis

All the steps were performed at room temperature. Three-day differentiated C25Cl48 cells cultured in collagen coated dishes were fixed with 2% paraformaldehyde (PFA; Sigma-Aldrich) for 5 min, washed with PBS/0.5%BSA and permeabilized in PBS/0.25% Triton X-100 for 5 min. After washing, cells were incubated with mouse monoclonal anti-troponin T antibody (1/100) for 1 h, then washed for three times and incubated for 30 min with the Alexa 555-conjugated anti-mouse secondary antibody (1/1000) and Phalloïdin iFLUOR 488 (1/1000) (Abcam) that binds to F-actin. The samples were washed two times and nuclei were counterstained with DAPI (final concentration of 0.1 mg/ml) for 1 min. Images (5–10 randomly selected nonoverlapping fields per coverslip for each condition) were acquired (10X objective) with a Zeiss Axio imager M1 epifluorescence microscope (Carl Zeiss SAS-Microscopy) and the ZEN imaging software. The morphological analyses were performed using the Fiji software as recently detailed [29]. DAPI-positive cells were analysed to determine the number of nuclei per image normalized to the total image area to compare all conditions. The red (Troponin-T) and blue (nuclei) channels for a given field of view were opened individually and thresholded (the same threshold for a given channel was applied to all images) to determine the average area of troponin-T-stained myotubes and the myogenic fusion index. The fusion index was calculated as the percentage of nuclei in troponin-T-positive multinucleated myotubes compared to the total number of nuclei visible in the image frame.

### Statistical analyses

Data were analyzed with the software Prism v9.4.1 software (Graph-Pad Software) and expressed as the mean ± SD. Sample number (n) indicates the number of independent biological samples in each experiment. Groups were compared with unpaired non-parametric multiple Mann–Whitney tests for all bar graphs shown in this study. Differences in means were considered statistically significant at p ≤ 0.05. For all graphs, n.s. means not significant, ^⁎^p ≤ 0.05, ^⁎⁎^p ≤ 0.01, ^⁎⁎⁎^p ≤ 0.001. For correlations, a simple linear regression between two groups was performed to obtain the R-squared value.

## Results

### Depletion of eIF4A3 or Y14 affects splicing in the 3’end of the Dp427m transcript

**Fig. 1:**
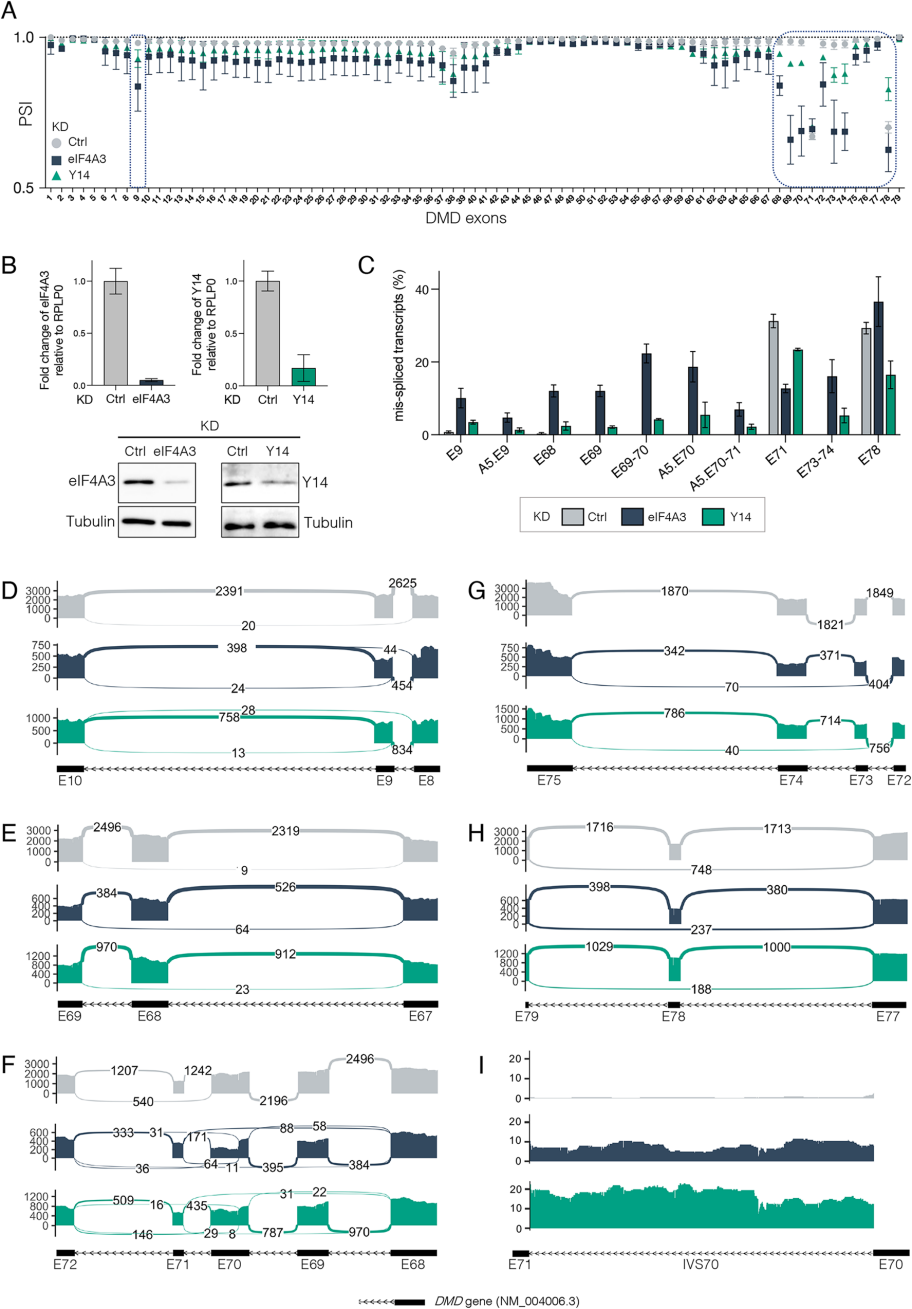
Splicing profile of the Dp427m isoform upon depletion of the EJC proteins eIF4A3 and Y14 .**A** Inclusion level of the 79 DMD exons **(**Percent Spliced In, PSI) calculated from *DMD*-targeted RNA-seq datasets after eIF4A3 (dark blue) or Y14 (green) KD (n = 2) in the C25Cl48 cell line differentiated for 3 days compared to control (Ctrl, grey) (n = 4). **B** eIF4A3 and Y14 mRNA and protein levels were monitored by RT-qPCR (upper panel, means ± SD, n = 2) and western blot analysis (representative images, bottom panel). **C** Mis-spliced transcripts identified with the IPSA software in eIF4A3 KD are depicted in the bar graph and compared to levels in Y14 KD and Ctrl conditions. **D**–**I** Sashimi plots color-coded by condition (Ctrl, eIF4A3, Y14 KD) depicting **(D**–**H)** the differentially spliced exons and **(I)** intron 70 (IVS70) retention. The raw junction read counts (mean of duplicates) are shown on top of each junction represented as curved lines. Schematic of the primary transcript (ENST00000367455) for the DMD gene from the Ensembl database, version 75, with exons shown as boxes, introns shown as lines, and arrows indicating the direction of transcription

To determine whether the EJC plays a role in the splicing regulation of the Dp427m transcript, we analyzed the splicing changes induced by the depletion of the two EJC core components eIF4A3 and Y14 in the human C25Cl48 muscle cell line using 454 amplicon sequencing (Fig. 1A). This DMD-targeted RNA-seq approach, which consists of sequencing the entire cDNA (11.3 kb) amplified as a single fragment by long-range PCR, is well suited for in-depth analysis of the lowly expressed DMD gene [23]. The transfection of either control siRNA (Ctrl KD) or siRNAs targeting eIF4A3 or Y14 was performed in proliferating cells that were cultured for 3 additional days in differentiation medium to allow expression of both Dp427m RNA and dystrophin protein [24]. The knockdown efficiency was verified by RT-qPCR and western blotting (WB) (Fig. 1B). Sequencing data from two independent biological replicates of eIF4A3 KD and Y14 KD were analyzed in comparison to four control replicates. Exon-inclusion levels, given as PSI values, were computed for the 79 annotated exons of the Dp427m isoform using the exon-centric metrics of the IPSA software (Fig. 1A and Supplementary Table 2). Of note, the basal level of alternative splicing for exons 71 and 78 in the C25Cl48 cell line [24] can be seen in the control condition. Except for exon 9, the KD of eIF4A3 and to a lesser extent that of Y14, selectively affected the inclusion of a cluster of exons at the 3’end of the transcript (from exon 68–78). A decrease in exon inclusion may result from multiple splicing events that are more complex than single exon skipping. Differential splice junction (SJ) usage analysis between EJC KD and the control condition was conducted with the IPSA intron-centric metrics to best characterize the splicing events involved. By selecting a change in junction usage above 5% (|∆SJ|≥ 0.05) in the eIF4A3 KD condition, 10 different splicing events were identified consisting in single or double exon skipping and use of an alternative 5’splice site (A5) in exon 9 (A5.E9) and in exon 70 (A5.E70) (Fig. 1C and Supplementary Table 2). Unexpectedly a decrease in exon 71 skipping was noticed (Fig. 1C) that was not consistent with the unchanged PSI value reported in Fig. 1A. We were able to determine that the proportion of transcripts lacking exon 71 was indeed the same, but that there was a redistribution between transcripts with a single skipping of exon 71 and new larger variants excluding exon 71 (i.e. A5.E70–71). The heterogeneous population of alternative transcripts involving exon 71 has been further characterized below. Upon depletion of Y14 similar splicing events were generally present at a lower level, except for exon 78, which showed a strong response in the opposite direction (exon inclusion) to that observed in eIF4A3 KD. We also looked for intron retention events by focusing on small DMD introns whose detection was compatible with the amplicon approach used. Analysis of read coverage of the 9 shortest introns, less than 1 kb in length, disclosed a low level of retention of intron 70 (698 bp). Representative splicing patterns and junction reads for all detected aberrant splicing events are depicted in Sashimi plots in Fig. 1D–I.

**Fig. 2:**
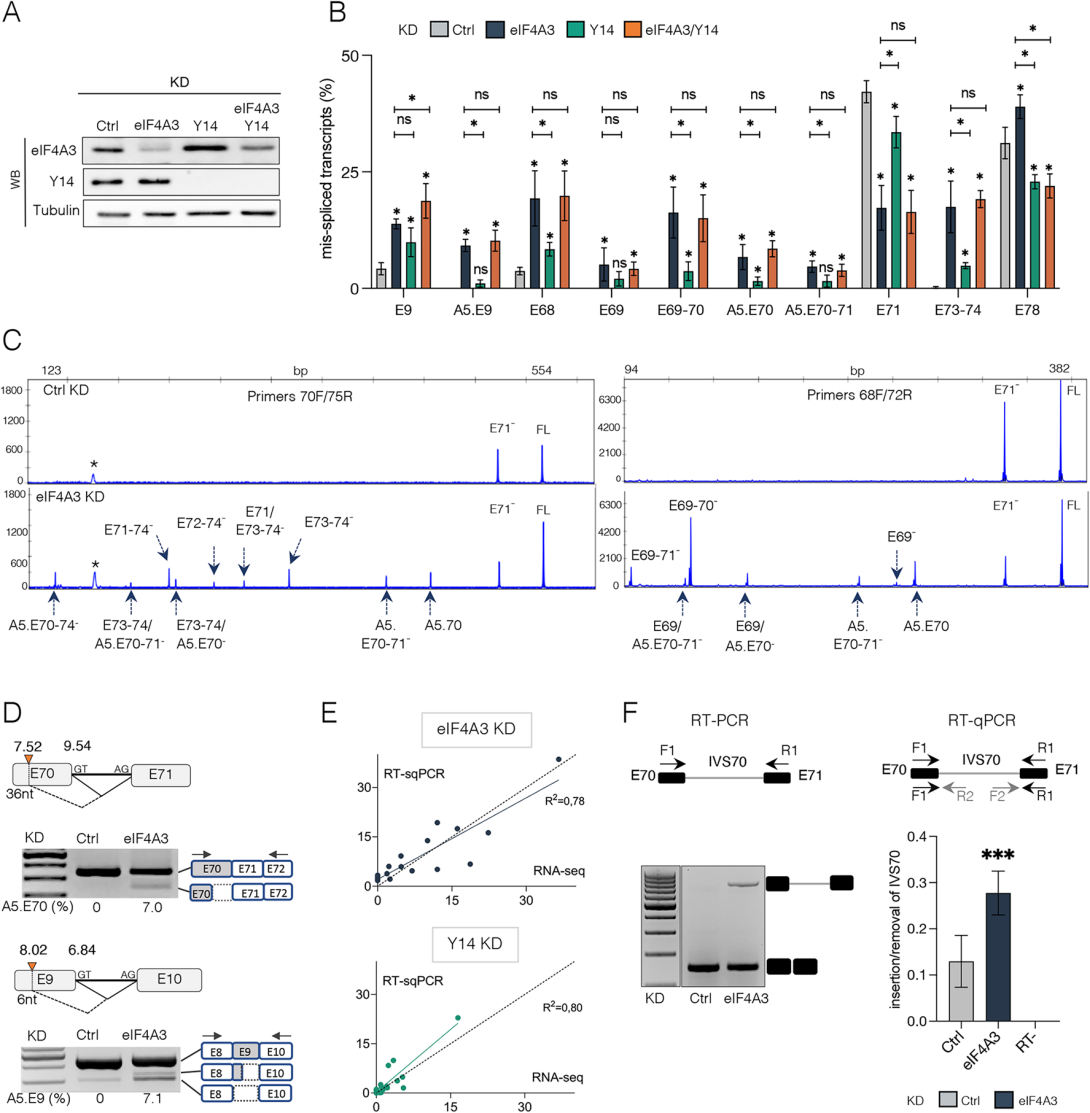
Validation of DMD aberrant splicing events upon depletion of EJC components. **A** Western blot analysis of eIF4A3, Y14 and Tubulin as loading control in the different KD conditions. **B** Quantification of splicing events in Ctrl, eIF4A3 and/or Y14 KD by fluorescent semi-quantitative RT-PCR (QFPCR) (n = 4) in C25Cl48 cells. Data are shown as means ± SD (*p < 0.05; multiple Mann–Whitney test). ns, non-significant. **C** Representative electropherograms (x-axis: fragment size; y-axis: fluorescence intensity) of capillary electrophoresis analysis of large-sized amplified fragments using primers 70F/75R or 68F/72R in eIF4A3 KD compared to control (Ctrl). Size-calling of splicing events (i.e*.* exon 71 skipping (E71^-^), full-length RT-PCR product (FL)) was performed with the GeneMapper^®^ v6.0 Software (arrows). (*) Non-specific band. **D** Representative agarose gels showing activation of alternative donor splice site (orange triangle) in exon 70 (A5.E70) and in exon 9 (A5.E9) in eIF4A3 KD condition (values in % below the gel). The identified splicing patterns are shown schematically at the top of the gels with the MaxEnt scores (in bold) for the natural and alternative donor splice sites. On the right side of the gels, gray and empty dotted boxes represent included and deleted exonic sequences, respectively. Black arrows, position of the primers. **E** Correlation between DMD-targeted RNA-seq and QFPCR data in eIF4A3 and Y14 KD. Plots for linear regression analyses and the R-squared (R^2^) values are shown. **F** Validation of IVS70 (698 bp) in eIF4A3 KD condition by RT-PCR using primers in exons 70 and 71 (F1/R1) (left panel) or RT-qPCR using three different primer pairs (F1/R1, F1/R2, F2/R1) (right panel). In qPCR, ratios of relative expression of DMD transcript with intron retained (mean of data (n = 8) measured with primers F1/R2 (n = 4) and F2/R1 (n = 4)) compared to relative DMD transcript expression with intron removed (measured with primers F1-R1 (n = 4)) are shown. No reverse transcriptase control (RT-). Data are mean ± SD (***p < 0.001; multiple Mann–Whitney test). All measurements are normalized to RPLP0 expression

To support the targeted RNA-seq data, we validated the effects of eIF4A3, Y14 or the double eIF4A3/Y14 KD on DMD splicing from independent knockdown experiments (n = 4). An efficient knockdown was achieved at RNA and protein level in all conditions (Fig. 2A and Supplementary Fig. 1A). Here, the splicing changes were detected by semi-quantitative fluorescent RT-PCR (QFPCR) using primers in constitutive exons that flank splicing events (Fig. 2B and Supplementary Fig. 2) as well as by conventional PCR for simple events (Supplementary Fig. 1B). We validated all 10 splicing changes previously detected by targeted RNA-seq, including the activation of the two alternative 5’ss leading to inclusion of shortened exon 9 (6 nt) and exon 70 (36 nt) in the mature transcripts (Fig. 2B–D). The identity of the splicing events was verified by Sanger sequencing (Supplementary Fig. 3). As previously observed, knockdown of Y14 had a more modest effect on DMD splicing. The splicing profile in the double KD cells was superimposable to that obtained upon eIF4A3 KD except for exon 78 which showed a behavior similar to that of Y14 KD (Fig. 2B). Fluorescent PCR capillary electrophoresis, which can discriminate single-nucleotide differences in PCR products, enabled us to refine the inventory of aberrant splicing events present in the 3’ end of the Dp427m transcript. Analysis of larger regions of DMD transcripts using primer pairs located in exons 68/72 and 70/75 revealed the complexity of mis-spliced transcripts that accumulate upon depletion of EJC components (Fig. 2C). These findings extended the list of EJC-dependent splicing changes to 18 (Supplementary Table 3). Many of them are associated with the use of the A5.E70, albeit of lower strength than the natural 5’ss splice site (MaxEnt 7.52 and 9.54, respectively) (Fig. 2D). We found 4 of these additional events by re-examining the RNA-seq data at a level below the 5% threshold considered for data analysis (Supplementary Table 2, 0.05 >|Δ SJ|≥ 0.01).

Overall, strong correlations were observed between RT-QFPCR and targeted RNA-seq data for splicing analyses in eIF4A3 KD (R^2^ = 0.78) and Y14 KD (R^2^ = 0.80) conditions (Fig. 2E, Supplementary Fig. 4A). We also independently validated the retention of intron 70 (IVS70) in eiF4A3 KD by standard RT-PCR and agarose gel electrophoresis (Fig. 2F, left panel) and quantification by RT-qPCR using three different primer sets to amplify the correctly spliced E70–E71 junction (F1-R1) or specifically the intron-retaining (F1-R2 and F2-R1) transcripts, which showed a significantly higher level of intron-retained transcripts compared with the control (Fig. 2F, right panel). Overall, our findings support a role for EJC in the efficiency and fidelity of splicing of the DMD pre-mRNA.

### Deregulation of DMD splicing is not due to repression of EJC-dependent NMD

**Fig. 3:**
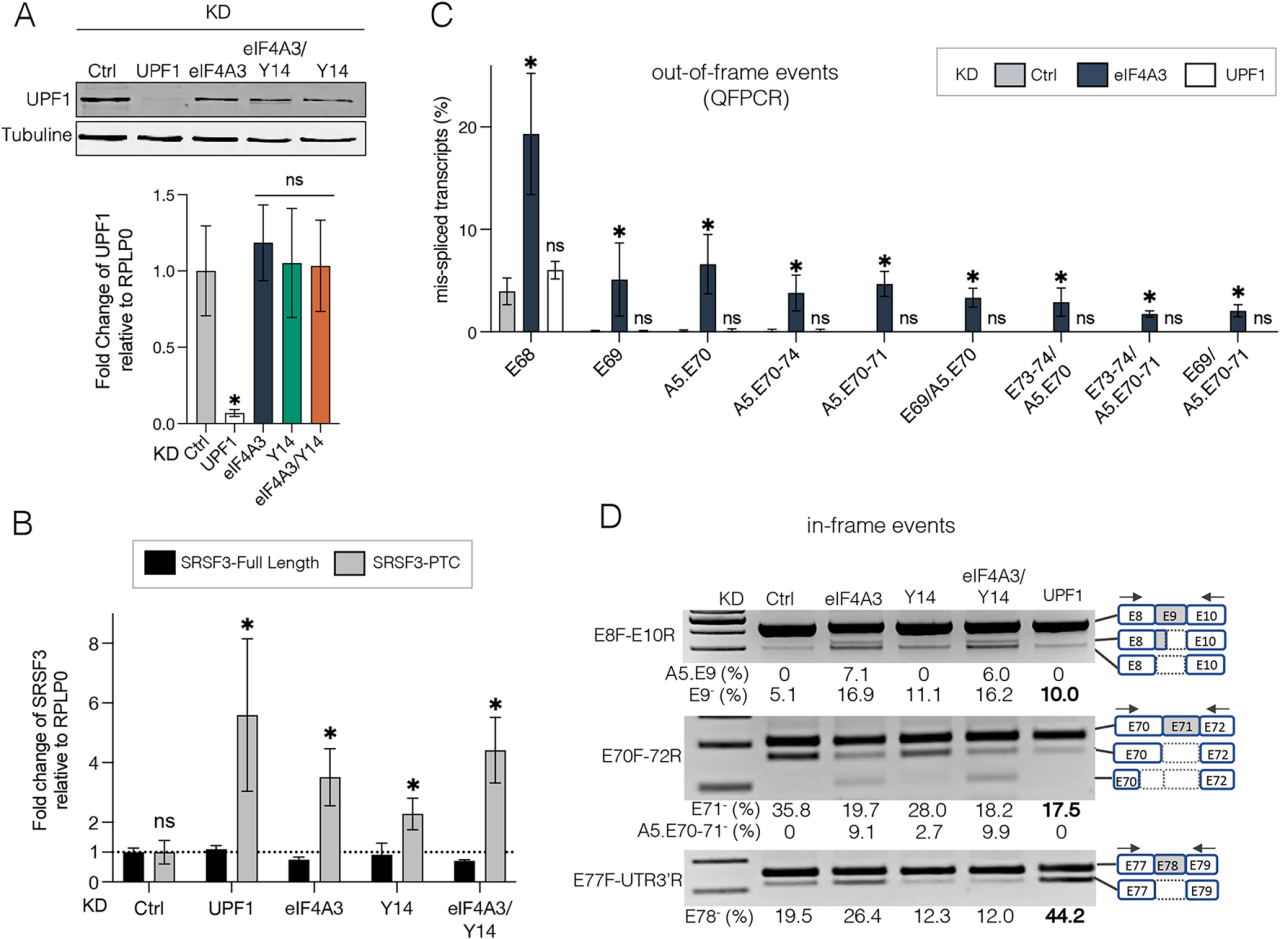
Effect of UPF1 KD on EJC-dependent splicing events. **A** Assessment of UPF1 expression in UPF1, eIF4A3, Y14 and Ctrl KD conditions at protein and mRNA levels by western blot using Tubulin as loading control (upper panel) and RT-qPCR (bottom panel), respectively. **B** RT-qPCR of the SRSF3 NMD-sensitive transcript isoform (SRSF3-PTC containing exon 4) compared to full-length SRSF3 isoform (SRSF3-Full Length) in Ctrl, UPF1, eIF4A3 and/or Y14 KD. **C** Bar graph showing levels of out-of-frame splicing events determined by QFPCR in UPF1 KD compared to eIF4A3 KD and Ctrl condition. In (**A**, **B**, **C**) all data are mean ± SD of 4 independent replicates (*p < 0.05; multiple Mann–Whitney test). ns, non-significant. **D** Representative agarose gels showing changes in the inclusion level of in-frame DMD E9, E71 and E78 upon UPF1 KD. The structure of splicing events is depicted on the right side of the gels and levels (%) of aberrant splicing events are given below the gel

In mammalian cells, EJC deposition is pivotal for the process of nonsense-mediated mRNA decay (NMD) that recognizes and degrades aberrant transcripts carrying premature termination codons (PTCs). The EJC serves as a binding platform for the NMD factors, Up-frameshift protein UPF1, UPF2 and UPF3b [30]. Thus, the detection of PTC-containing mRNAs in eIF4A3 KD and Y14 KD might as well be due to a less efficient EJC-dependent NMD instead of revealing a true role in splicing. To distinguish between these possibilities, we depleted the essential NMD factor UPF1 by siRNA in the C25Cl48 cells and compared the DMD splicing profile with that obtained in eIF4A3- and Y14-depleted cells. UPF1 mRNA and protein levels were efficiently downregulated and we could verify that UPF1 expression was not affected in previous eIF4A3 and Y14 KD experiments (Fig. 3A). Decrease in NMD efficiency was assessed by quantifying the NMD-sensitive splice variant of the SRSF3 gene that includes the PTC-containing exon 4 (SRSF3-PTC) [31]. As expected, UPF1 KD led to stabilization and an increase in the detected level of the SRSF3-PTC isoform relative to the control, while the level of the SRSF3 isoform encoding the full-length protein remained unchanged (Fig. 3B). Consistent with the role of EJC in NMD activation, the SRSF3-PTC isoform was also found to escape degradation to some extent in EJC KD.

Importantly, the 9 DMD out-of-frame splice variants caused by eIF4A3 KD could not be detected by QFPCR in UPF1-depleted cells (Fig. 3C), indicating that a decrease in NMD efficiency likely does not account for the presence of these PTC-containing transcripts in eIF4A3 KD cells. The presence of NMD-insensitive in-frame splice variants generated by eIF4A3 and Y14 KD is a further indication of the role of EJC components in DMD splicing. As expected, most of these in-frame splicing events were not found in UPF1-depleted cells with the exception of three exons (Fig. 3D and Supplementary Fig. 5B). A modest increase in exon 9 skipping compared to control (10% vs. 5%) was indeed detected as well as significant variations in the inclusion level of the short exons 71 (39 bp) and 78 (32 bp). The 50% decrease (from 35.8% to 17.5%) in exon 71 skipping was confirmed by QFPCR analysis of the large PCR fragment (primers E70F–75R), which also ruled out the presence of additional complex splicing events including exon 71 as previously observed for eIF4A3 KD (Supplementary Fig. 5C). For exon 78, exon skipping level was greatly increased (44.2%) compared with control (19.5%) and eIF4A3 KD (26.4%). Skipping of the penultimate exon 78 shifts the reading frame in the last exon 79. A more downstream stop codon is used that modifies the C-terminal region of the dystrophin protein, replacing the last 13 amino acids by 31 new ones [32]. Marked deregulation of exons 71 and 78 splicing was also observed when UPF2, another core NMD factor, was depleted (Supplementary Fig. 5D). Our findings therefore suggest that the NMD factors UPF1 and UPF2 may play a direct or indirect role in the splicing of these *DMD* exons. This is in line with splicing alterations previously reported in other genes upon depletion of UPF1 alone or coupled to EJC components [10, 33, 34] that suggest a role for core NMD components in splicing.

### Wild-type eIF4A3 and Y14 proteins selectively rescue EJC siRNA-induced splicing defects in DMD mRNA

**Fig. 4:**
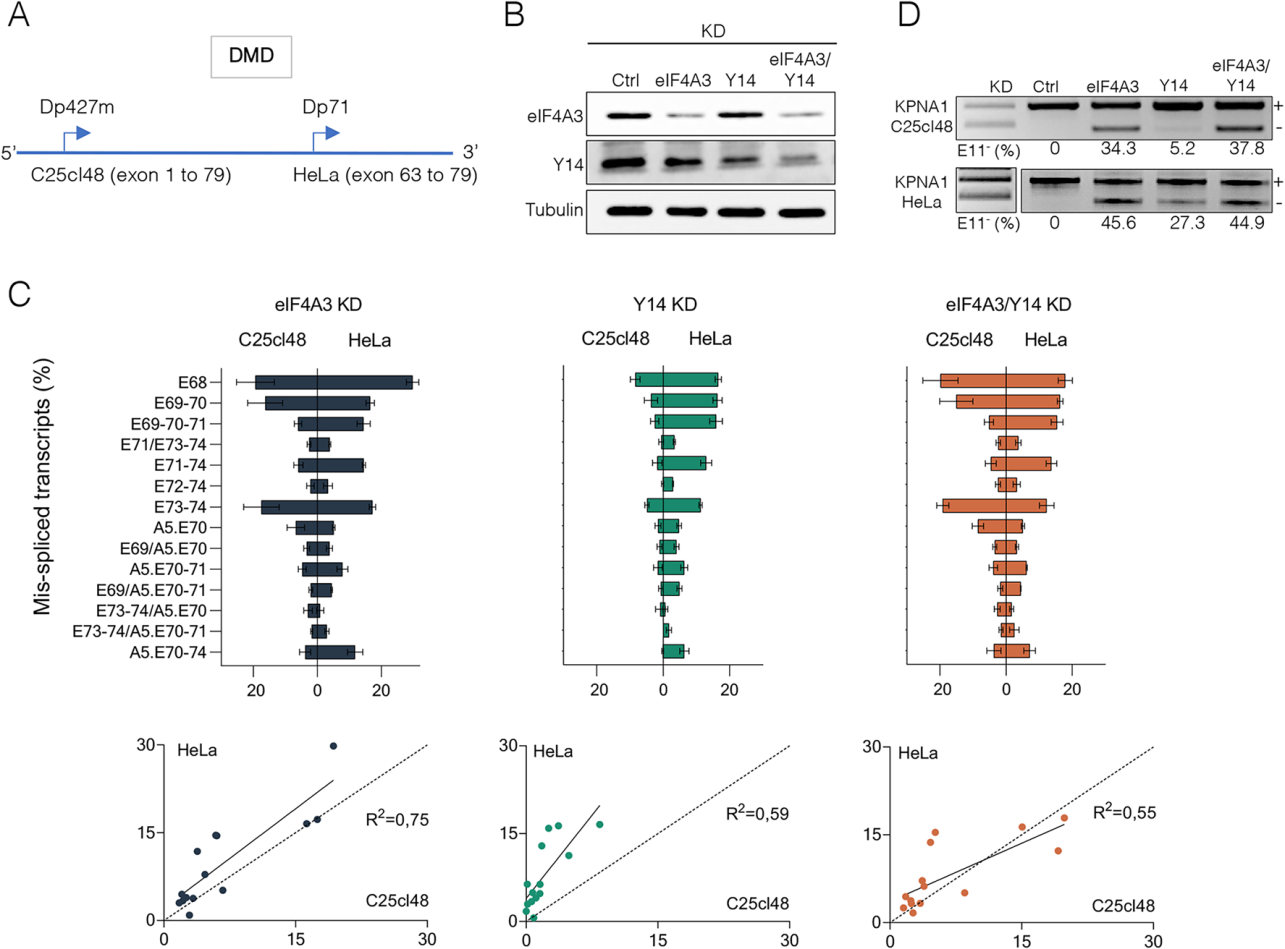
HeLa cell line as a model to study EJC-dependent splicing events in the 3’ end of DMD mRNA. **A** Scheme depicting the C25Cl48 and HeLa cells specific DMD isoforms (Dp427m and Dp71, specific promoter position). **B **Representative western blot showing siRNA-mediated knockdown of eIF4A3 and/or Y14 in HeLa cells with Tubulin as a loading control. **C** The splicing events quantified by QFPCR induced by eIF4A3 and/or Y14 KD in HeLa cells (Dp71) are correlated with those detected in C25Cl48 cells (Dp427m). Plots for linear regression analyses and the R-squared (R^2^) values are shown below the bar graphs. **D** Agarose gels of RT-PCR of KPNA1 exon 11 comparing exon skipping level (E11^-^, %) in eIF4A3 and Y14 KD in C25Cl48 and HeLa cells

We next assessed the ability of siRNA-resistant wild-type (WT) FLAG-tagged eIF4A3 and Y14 proteins (eIF4A3-WT and Y14-WT) or EJC-binding incompetent mutant version of eIF4A3 (eIF4A3-Mut) to rescue splicing changes induced by eIF4A3 and Y14 depletion. These experiments were performed in HeLa cells as optimal conditions for RNAi rescue experiments could not be successfully established in the C25Cl48 cells. In HeLa cells, a short dystrophin isoform (Dp71) is expressed from an alternative promoter located in intron 62 that includes all downstream exons (exons 63–79) of the Dp427m isoform (*i.e.* all deregulated exons in eIF4A3 KD except exon 9) (Fig. 4A). First, we validated that the KD of eIF4A3 and Y14 in HeLa cells caused splicing changes in Dp71 similar to those observed in the Dp427m isoform in C25Cl48 cells. The KD efficiency was checked by western blotting (Fig. 4B). It should be noted that the high basal level of exon 78 skipping in HeLa cells makes it difficult to study its skipping under KD conditions (Supplementary Fig. 6A, B). Overall, the Dp71 in HeLa cells showed the same deregulated splicing events upon EJC components KD as Dp427m in C25Cl48 cells, with a good correlation in the level of aberrant splicing events in eIF4A3 KD between the two cell lines (Fig. 4C). Unlike in C25Cl48 cells, the Y14 KD splicing pattern in HeLa cells achieved higher level of mis-spliced transcripts, being closer to that of the eIF4A3 KD. This difference between the two cell lines was not limited to the *DMD* gene and was also visible for exon 11 of the control gene KPNA1 [10], reflecting cell type-specific behavior in response to changes in the intracellular level of EJC components (Fig. 4D).

**Fig. 5:**
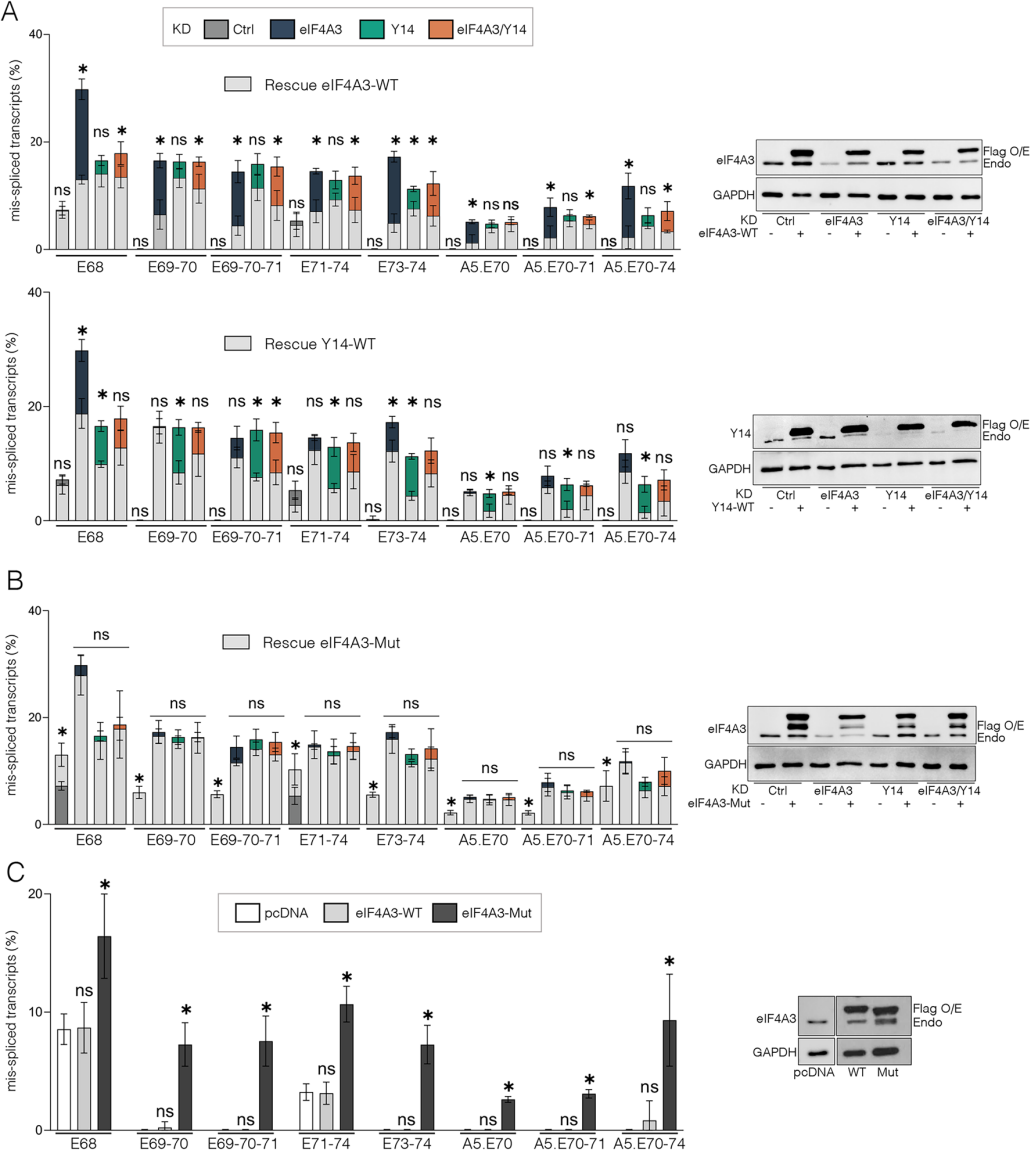
eIF4A3 and Y14 individually contribute to the inclusion of DMD exons but EJC formation is required for correct DMD splicing. **A** HeLa cells depleted for eIF4A3 and/or Y14 were transfected with siRNA-resistant wild type (WT) eIF4A3 (upper panel) and Y14 (bottom panel) expression vector with a FLAG tag or **B** with a mutant (Mut) siRNA-resistant eIF4A3 that does not form EJC (eIF4A3-Mut). Histograms show the quantification by QFPCR of mis-spliced transcripts in rescue conditions (light grey bars) compared to eIF4A3, Y14, eIF4A3/Y14 and Ctrl KD conditions. Data are mean ± SD (n = 4) (*p < 0.05; multiple Mann–Whitney test). ns, non-significant. **C** The wild-type (WT) or mutant (Mut) siRNA-resistant FLAG-eIF4A3 protein was overexpressed in untreated HeLa cells and splicing events were quantified as described above and compared to the control condition (empty pcDNA vector, white bars). Detection of endogenous (endo) and FLAG-tagged (Flag) eIF4A3 or Y14 proteins by western blot is shown in the right panel. GAPDH serves as loading control. The additional upper band on western blot detecting the exogenous eIF4A3-Mut protein can correspond to different forms of the proteins

Wild-type eIF4A3, Y14 or mutant eIF4A3 constructs were transiently transfected in HeLa cells to complement eIF4A3 and Y14 KD. Western blot analysis was used to monitor the expression of exogenous FLAG-tagged proteins in these experiments (Fig. 5A, B, right panels). Except for two splicing events (skipping of E68 and E73–74), we found that eIF4A3-WT expression, but not Y14-WT, was able to significantly rescue si-eIF4A3-induced splicing events back to control levels (Fig. 5A, upper and bottom panels, light grey bars stacked on dark blue bars) and conversely, the Y14-WT construct rescued si-Y14-induced splicing events but not eIF4A3-WT (Fig. 5A, lower and bottom panels, light grey bars stacked on green bars). In double KD cells, transfection of either WT proteins resulted in an intermediate level of rescue, which was most often significant with eIF4A3-WT (Fig. 5A, light grey bars stacked on orange bars). Ectopic expression of eIF4A3-Mut, which lacks the ability to bind to the MAGOH-Y14 heterodimer and prevents the formation of the complex, was unable to reverse the effects of eIF4A3 KD (Fig. 5B, upper panel, light grey bars stacked on color bars). Furthermore, its expression in untreated cells induced, to some extent, the aberrant splicing events present in eIF4A3 KD, most likely by exerting a dominant negative effect on the endogenous wild-type eIF4A3 protein (Fig. 5C). Taken together, these data show that eIF4A3 and Y14 individually play a role in the regulation of DMD pre-mRNA splicing and that this regulation is dependent on EJC assembly.

.

### EJC peripheral protein complexes modulate splicing of DMD exons 71 and 78

**Fig. 6:**
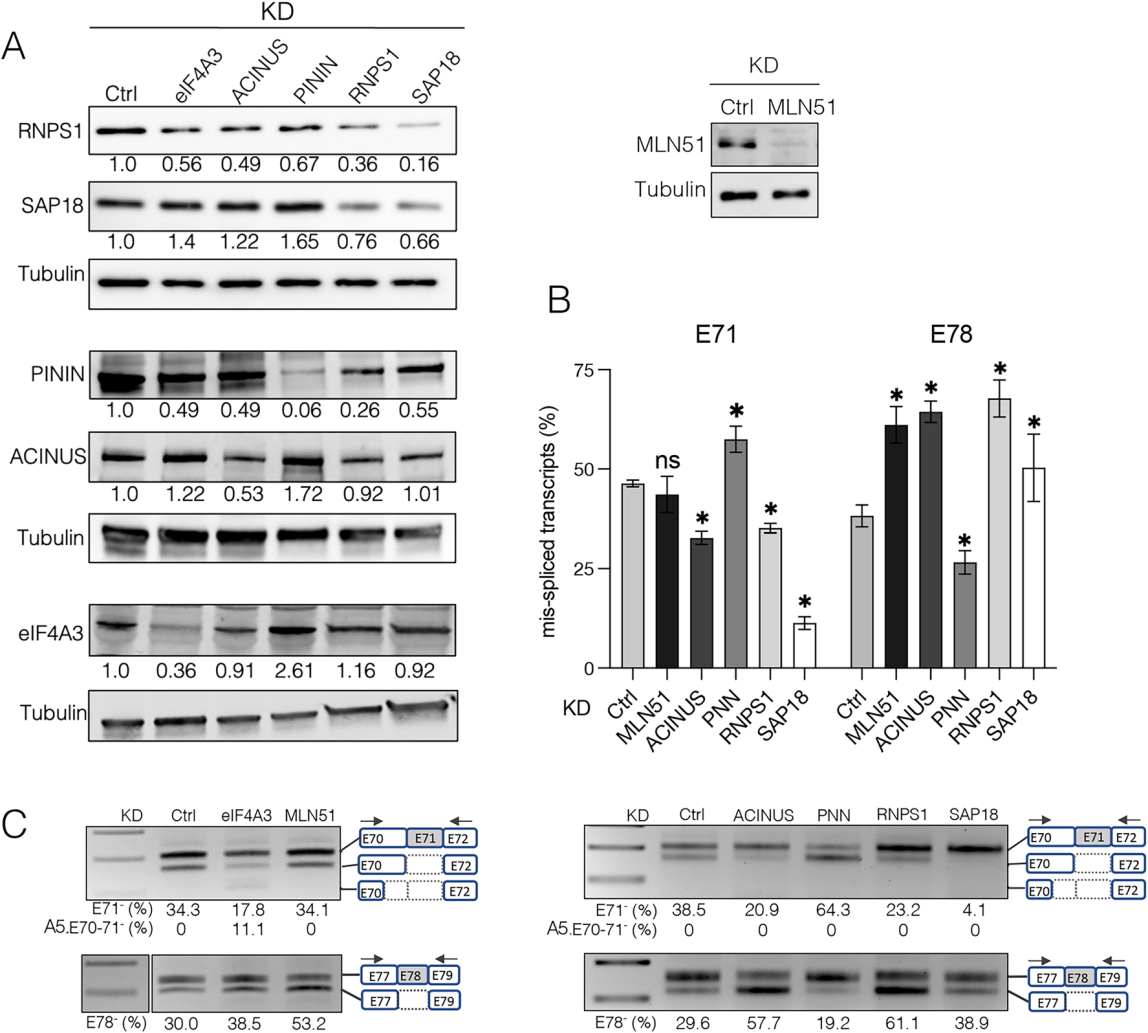
The EJC-peripheral proteins regulate splicing of DMD exons 71 and 78. **A** Western blot analyses of C25Cl48 cells depleted for eIF4A3 and proteins of the ASAP/PSAP complexes. Tubulin was used as a loading control (left panel). Western blot showing the KD efficiency of MLN51 (right panel). **B** Quantification by QFPCR of exon 71 (E71) and exon 78 (E78) skipping level (%). Data are mean ± SD (n = 4) (*p < 0.05; multiple Mann–Whitney test). ns, non-significant. **C** Representative agarose gels showing changes in the inclusion level of E71 and E78 (%, skipping level below the gels) in KD of MLN51 and ASAP/PSAP components (ACINUS, PININ, RNPS1 and SAP18)

To better understand how EJC influences DMD pre-mRNA splicing, we determined whether peripheral protein MLN51 and complexes ASAP/PSAP play a role in this regulation. ASAP and PSAP are composed of RNPS1 and SAP18 and differ by one of the two specific proteins, namely ACINUS and PININ for ASAP and PSAP, respectively. We performed siRNA-mediated depletion of each component of the ASAP/PSAP complexes and of MLN51 in C25Cl48 muscle cells. The efficiency of siRNAs and possible cross effects on the expression of other proteins in the complexes were assessed by RT-qPCR (Supplementary Fig. 7A, B) and western blot analysis (Fig. 6A). Good to moderate silencing efficiency of siRNAs was generally achieved. An increase in SAP18 mRNA level under all siRNA conditions (Supplementary Fig. 7A) was noticed as well as variations in the levels of proteins of the PSAP/ASAP complexes in various KD conditions. In particular, we observed an interdependence of RNPS1 and SAP18 protein levels and an increased SAP18, ACINUS, and eIF4A3 protein levels upon PININ KD (Fig. 6A). The expression of eIF4A3 protein was unchanged in ACINUS, SAP18 and RNPS1 KD. In contrast to the eIF4A3 and Y14 KD, only exon 71 and exon 78 were found to be strongly impacted upon MLN51 and ASAP/PSAP KD. As shown by QFPCR analysis (Fig. 6B) and agarose gels (Fig. 6C), exon 78 skipping was significantly increased in the KDs of MLN51 and ASAP components (ACINUS, RNPS1, SAP18) in contrast to PININ KD, which induced exon 78 re-inclusion. Conversely, for exon 71, PININ KD increased exon 71 skipping, whereas the KD of ASAP components, in particular SAP18, promoted its inclusion.

These data support a distinct functional role for ASAP or PSAP complexes in regulating specific splicing events, as previously reported [13]. The two alternative 5’ss in exon 9 and exon 70 were not detected upon RNPS1 depletion (Supplementary Fig. 7C), as might have been expected given its reported role in suppressing cryptic or reconstituted 5' splice sites [15]. Our data therefore show that peripheral EJC proteins play a distinct role from central EJC components on splicing in the 3' region of DMD transcripts, with each of the ASAP and PSAP complexes differentially and specifically promoting the inclusion of exon 78 or exon 71, respectively.

### Reduced expression of EJC factors impairs C25Cl48 myoblast differentiation

**Fig. 7:**
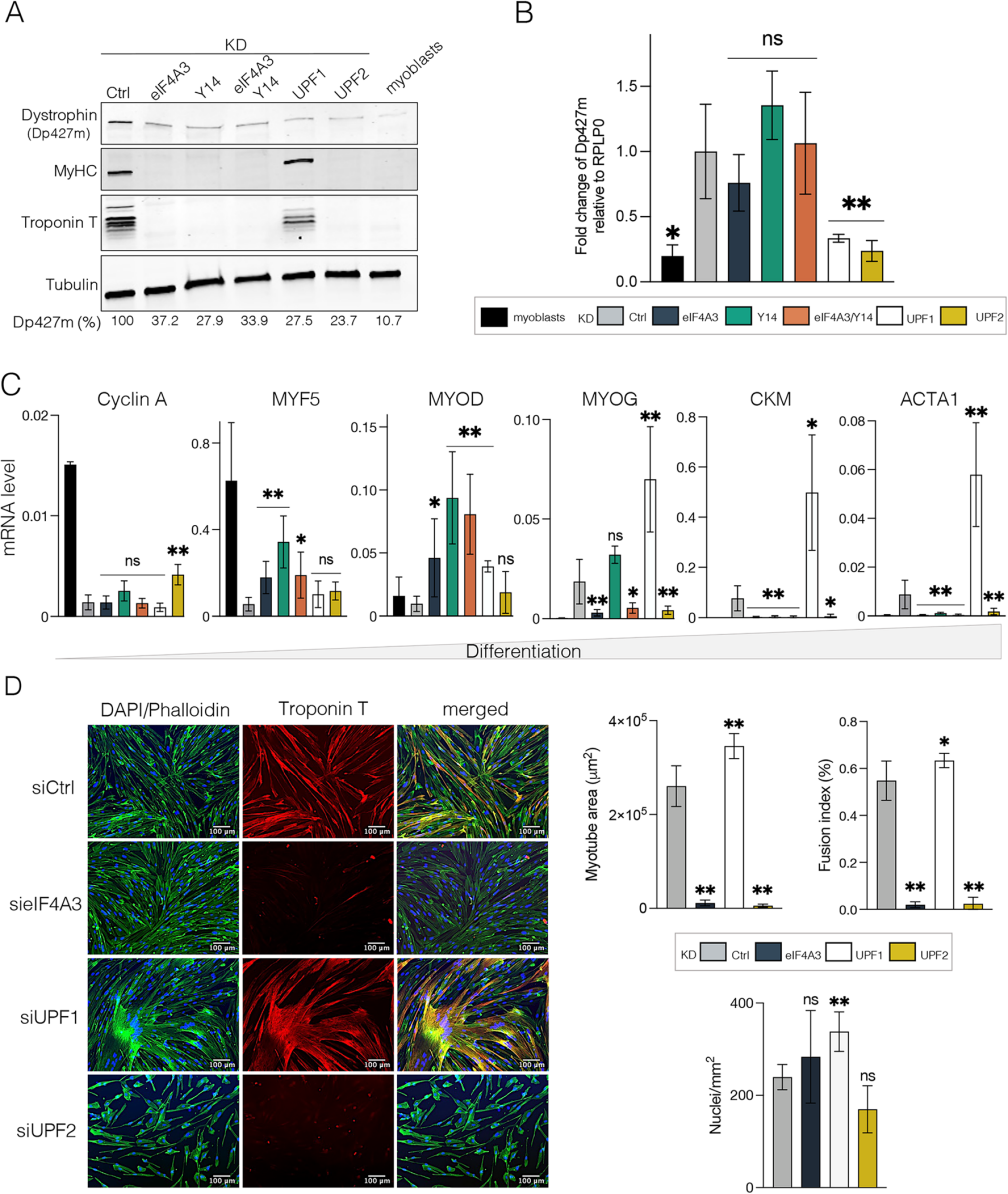
Depletion of EJC components in C25Cl48 cells decreases dystrophin expression and impairs myogenic differentiation. **A** The Dp427m expression level in C25Cl48 cells depleted for EJC (eIF4A3, Y14, eIF4A3/Y14), UPF1 and UPF2 was assessed by western blot in comparison to siRNA control (Ctrl) or untreated proliferating C25Cl48 cells (myoblasts). Myosin heavy chain (MyHC) and Troponin T serve as markers of the differentiation state and Tubulin as loading control. Quantification by RT-qPCR of **B** Dp427m mRNA and **C** the myogenic differentiation factors Cyclin A, MYF5, MYOD, MYOG, ACTA1, CKM. Data are mean ± SD (n = 5) (*p < 0.05; **p < 0.01; multiple Mann–Whitney test). ns, non-significant. **D** Representative images of immunofluorescence microscopy at 10X of C25Cl48 cells treated with siRNAs (Ctrl, eIF4A3, UPF1 and UPF2) for Troponin-T (red), Phalloïdin-488 to visualize actin filaments (green) and DAPI (blue). Scale bar = 100 µm. Myotube morphology was characterized by calculating mean myotube area, total number of nuclei and fusion index (nuclei per myotube/total nuclei). (*p < 0.05; **p < 0.01; multiple Mann–Whitney test). ns, non-significant

Finally, we monitored the consequences of the depletion of EJC and NMD components on the Dp427m expression at the mRNA and protein levels. The C25Cl48 muscle cells were treated with control, eIF4A3, Y14, UPF1 and UPF2 siRNAs and harvested after 3 days of differentiation as before. Consistent with the increased expression of dystrophin during differentiation, observed notably in C25Cl48 cells [24], a higher Dp427m mRNA and protein level was detected in KD control cells differentiated for 3 days, compared with myoblasts (Fig. 7A, B). Intriguingly for each other siRNA condition tested, the WB showed a decrease in Dp427m protein expression to a residual level of around 30% of the control level, a level nevertheless higher than that of untreated undifferentiated myoblasts (10.7%). This was correlated with a significantly lower level of Dp427m mRNA for both UPF1 and UPF2 NMD factors, whereas mRNA level was unchanged in eIF4A3 and/or Y14 KD conditions (Fig. 7B). During muscle differentiation, an isoform switch occurs between Dp71, which predominates in myoblasts, and Dp427m, expressed in myotubes [35, 36]. Dp71 isoform expression remained low and not significantly different from control for all KD conditions, except for a slight increase in KD UPF2 (Supplementary Fig. 8). The reason for the decrease in Dp427m protein and mRNA levels in UPF1 KD is unclear. With regard to eIF4A3 and Y14 KD conditions, we can hypothesize that the decrease in dystrophin protein level could in part be explained by the translation of out-of-frame transcripts, stabilized due to reduced NMD efficiency (Fig. 7B), giving rise to an abnormal, possibly unstable protein.

Since Dp427m protein levels increase during skeletal muscle differentiation, we also wondered whether its decrease in EJC-depleted muscle cells could be the result of an alteration in this process. We therefore sought to further characterize the differentiation state of the cells in the different KD conditions compared to control KD and untreated myoblasts. Western blot analysis showed that troponin T and myosin heavy chain (MyHC), two myogenic markers expressed in multinucleated myotubes, were expressed similarly to the control siRNA in UPF1 KD but was completely absent in eIF4A3, Y14 or UPF2 KD (Fig. 7A). We profiled the expression of muscle-specific marker genes by RT-qPCR (Fig. 7C). Cyclin A, which is essential for cell cycle progression in proliferating cells, was detected only in myoblasts and, to a lesser extent, in UPF2 KD, indicating cell cycle arrest in the other KD conditions, as occurs before differentiation. However, the analysis of the myogenic commitment factors MYF5 and MYOD, the early differentiation factor Myogenin (MYOG) as well as the muscle-specific proteins expressed at a later stage of differentiation, actin alpha 1 (ACTA1) and creatine kinase M-type (CKM), disclosed significant differences between these conditions. Compared to the control, C25Cl48 cells depleted for eIF4A3 and/or Y14 exhibited higher levels of MYF5 and MYOD and an absence or low level of MYOG, ACTA1 and CKM, a profile that would indicate a blockage in the first stages of differentiation. In contrast, and consistent with the described role of UPF1 in repressing myogenesis by promoting MYOD protein degradation [37], the depletion of UPF1 enhanced myogenesis and increased expression of MYOD, MYOG, ACTA1 and CKM differentiation markers (Fig. 7C). These results were confirmed by immunofluorescence studies that displayed significant morphological differences between KD conditions (Fig. 7D). Compared with the control, cells lacking eIF4A3, although elongated in shape, showed an almost complete absence of troponin staining and a very low myotube area as well as fusion index like UPF2 KD in which myoblasts are maintained in an undifferentiated state. In contrast, UPF1 KD cells presented a strong troponin T staining of the cells, a significant increase in cell surface area and fusion index. Overall, our results show that reduced expression of EJC factors alters skeletal muscle differentiation.

## Discussion

This study provides evidence that the EJC plays an important role in regulating splicing in the multi-exon DMD gene encoding the dystrophin protein in skeletal muscle, which causes a major genetic disease in humans when defective. Previous studies have reported a role of eif4a3 in ryanodine receptor pre-mRNA splicing and muscle cell function during early vertebrate embryogenesis in *Xenopus laevis* [38] or the functions of zebrafish Rbm8a and Magoh proteins in muscle and neural lineages [39], but there is a lack of data on the contribution of EJC to splicing regulation in human skeletal muscle. Here, we show that reduced expression in the human C25Cl48 muscle cell line of the two EJC core components, eIF4A3 and Y14, affects splicing accuracy in the DMD gene and leads to numerous aberrant splicing events. UPF1 depletion experiments verified that this pattern was not the result of a reduction in the degradation efficiency of PTC-bearing transcripts and is therefore independent of the role of EJC in NMD.

Although EJCs are thought to be present on more than 90% of canonical exon-exon junctions [40], aberrant splicing events were remarkably non-randomly distributed, accumulating towards the 3′ end of the transcript in eIF4A3 and Y14 KD. The cascade of events gave rise to a complex splicing pattern resembling the transcript disintegration previously reported for other genes [15]. The same exons were found to be deregulated in eIF4A3 and Y14 KD in Dp427m and Dp71, albeit in different proportions and, in rare occasions, in opposite directions, as in the case of exon 78. Rescue experiments confirmed the specific and non-functionally interchangeable role of the two EJC components and the need for the complex to assemble.

The majority of the identified EJC-regulated splicing events are independent of the PSAP and ASAP complexes. Depletion of RNPS1, SAP18, PININ and ACINUS changed inclusion level of DMD exons 71 and 78 only. Expression data at mRNA and protein level suggest that these changes in splicing are more likely to be due to deregulation of the complexes than on the specific effect of one of the components. As previously reported [13], ACINUS and PININ may act in opposite ways through coordinated, possibly mutually exclusive, recruitment of the PSAP and ASAP complexes to the EJC to modulate the inclusion level of DMD exons 71 and 78. Supporting the recent findings that MLN51 is not an obligate component of the EJC core [41, 42], the depletion of MLN51 did not replicate the splicing pattern seen in eIF4A3 KD.

The splicing events detected in the KD of EJC core components are mainly single or multi-exon skipping events that likely result from re-splicing mechanism, although such events cannot be easily distinguished from a regular exon skipping. The reported protective role of EJC in suppressing nearby cryptic splice sites [16] was exemplified by the identification of two cryptic 5’ss that were de-repressed in exon 9 (A5.E9) and exon 70 (A5.E70). Activation of the A5.E70 contributes substantially to the mis-spliced transcripts detected in eIF4A3 KD. Despite a high MaxEnt score, the use of A5.E9 and A5.E70 has not been reported in physiological or pathological conditions. Their inhibition is independent of the PSAP complex, in particular of the SR-like protein RNPS1, whose major role in suppressing downstream re-splicing at cryptic or reconstituted 5′ ss has recently been well documented [15, 43]. Interestingly, we identified a non-canonical EJC (ncEJC) in exon 70 (data not shown), immediately upstream of A5.E70 from publicly available xRIPiT and CLIP-seq data [40, 41, 44]. Whether it may be involved in splicing regulation of exon 70 remains to be demonstrated, as the function of these potential ncEJCs has yet to be elucidated.

EJCs multimerize into high-molecular-weight complexes within which they are associated with many other proteins in the nucleus. In particular, the EJC interactome includes sequence-specific SR (mainly SRSF1, SRSF3, and SRSF7) and SR-like proteins, which contribute to EJC recruitment and promote packaging of spliced RNAs into compact RNP particles [41, 45, 46]. SR proteins are important regulators of pre-mRNA constitutive and alternative splicing. This raises the question of whether defective recruitment of SR proteins may have contributed in some way to the aberrant DMD splicing pattern observed upon depletion of EJC components. However, the splicing patterns identified here are significantly different from those obtained by KD of SRSF1 and SRSF2 in a previous study [24].

Importantly, our data were extended to a different cell line that expresses the shorter Dp71 isoform from DMD intron 62. This supports the view that deregulation of splicing at the 3′-end of the transcript in EJC-depleted cells is probably not related to transcript size or to parameters related to transcription kinetics, but rather to region-intrinsic properties. It is worth noting that the exon–intron architecture of the 3’ region of the gene is distinguished from the rest of the gene by the presence of small introns (average size 5 kb versus 25 kb) that are spliced slowly and non-sequentially [47]. Among them, the 698 bp intron 70, which is retained to some level in eIF4A3 KD. This intron contains an alternative poly-adenylation site that is activated in Dp71 mRNA to give rise to a shorter Dp40 isoform [48]. The gene architecture may play a role in the spatial subnuclear localization and regulation of splicing of genes [49]. The 3’end of DMD transcript is prone to physiological tissue- and cell-specific alternative splicing. The inclusion of exons 71–74 and 78 is highly variable depending on the cell type and DMD isoforms expressed, notably in brain and retina [50, 51] in contrast to muscle tissue, in which we have shown that all exons are included [23]. Exons 68–79 encode the cysteine-rich and C-terminal domains of dystrophin. These two domains are involved in essential protein–protein interactions with ß-dystroglycan at the plasma membrane, and with cytoplasmic syntrophins and dystrobrevin for muscle fiber maintenance and cell signaling functions [21]. Deregulation of alternative splicing in this region may affect the sub-cellular localization and cellular functions of isoforms or the stoichiometry of recruited signaling pathway effectors such as syntrophins [22, 52, 53]. The sensitivity of DMD exons 68–78 to abnormal dosage of EJC components may indicate increased EJC occupancy and/or demand for splicing precision in this highly regulated region of the DMD gene, which is conserved in all long and short isoforms of dystrophin [21]. No mapping data are currently available for the Dp427m isoform in muscle cells, which would enable these hypotheses to be investigated. Recent findings indicate that EJCs are loaded to nearly 100% of exon junctions in humans or Drosophila model [39, 40, 54]. The differential deposition of EJC between exon-exon junctions as a function of certain mRNA characteristics remains to be clarified [54]. Further work is needed to elucidate the parameters that contribute to post-transcriptional regulation by EJC.

Functional EJC components have been reported to be important in controlling complex cellular functions. A proper dosage of assembled EJCs is required for neural stem cell division, differentiation and brain development. Dysfunctions of core and peripheral EJC components cause abnormal cell cycle progression and apoptosis [55], defects in centrosomal organization and ciliogenesis in neural stem cell [56] and are implicated in several forms of neurodevelopmental disorders in humans [57–59]. A study in rat cardiomyocytes has reported that eIF4A3 is required for maintaining cardiac contractility and cell architecture [60]. It has also recently been reported that EJCs influence the accessibility of mRNA to the N6-methyladenosine (m6A) modification, a destabilizing mark on mRNA, involved in RNA metabolism and various biological including cell differentiation, brain development, tumorigenesis [61, 62]. A change in the transcriptomic distribution of m6A when EJC components are down regulated may contribute to the reported phenotypes.

Here, we report that normal expression of EJC components is required for myogenic differentiation in human skeletal muscle cells. We show that depletion of eIF4A3 blocks normal muscle differentiation of myoblasts into myotubes. UPF1 is known to repress human skeletal muscle differentiation by promoting protein ubiquitination and degradation of MYOD, a master regulator of myogenesis [37]. We observed that UPF1 KD led to enhanced differentiation of myoblasts into myotubes compared to the control condition. Despite being increased, our findings suggest that the differentiation process may nonetheless be somehow defective. While myogenic regulatory factors were strongly up-regulated in UPF1 KD, DMD gene expression, which normally increases during differentiation [24], was down-regulated at mRNA and protein level as in UPF2 KD. However, UPF2 KD cells are characterized by a complete absence of muscle differentiation, high level of cyclin A and Dp71 isoform suggesting that they are blocked in an immature stage in contrast to UPF1 KD. The reason for the decreased DMD protein and mRNA levels in UPF1 KDs is yet to be identified.

Down-regulation of EJC components is expected to impact the expression of all tissue-specific dystrophin isoforms, including the long Dp427 ones in striated muscle, the Dp260 in retina and the Dp140 and Dp71 in brain [22]. Of note, a higher incidence of cognitive impairment, neuropsychiatric symptoms and neurodevelopmental defects is reported in DMD patients with mutations affecting the expression of these shorter cerebral isoforms [63]. This opens new avenues of research aimed at investigating how variations in dosage of EJC components may affect the maturation of DMD transcripts and functions of dystrophin isoforms in the central nervous system.

In conclusion, this study identified EJC components as important regulators of dystrophin isoforms expression and skeletal muscle differentiation, further illustrating the importance of EJC in post-transcriptional regulation of gene expression in human cells.

### Supplementary Information

Below is the link to the electronic supplementary material.

Supplementary file 1 (PDF 1895 KB)

Supplementary file 2 (xlsx 67 KB)

Supplementary file 3 (PDF 83 KB)

Supplementary file 4 (PDF 100 KB)

## Data Availability

The datasets generated during and/or analyzed during the current study are available in electronic format in the Supplementary Information.
