# Supplementary material for: The exon junction complex is required for DMD gene splicing fidelity and myogenic differentiation
Source: Cell Mol Life Sci. 2024 Mar 21;81(1):150. doi: 10.1007/s00018-024-05188-1 (PMC10957711; doi:10.1007/s00018-024-05188-1)

Submitted to Cellular and Molecular Life Sciences

The Exon Junction Complex is required for DMD gene splicing fidelity and myogenic differentiation

Dylan Da Cunha[1], Julie Miro[1], Charles Van Goethem[2,3], Cécile Notarnicola[1], Gérald Hugon[1], Gilles Carnac[1], Mireille Cossée[1,2], Michel Koenig[1,2], Sylvie Tuffery-Giraud[1*].

- Corresponding author:

Sylvie Tuffery-Giraud
sylvie.tuffery@inserm.fr
[1]PhyMedExp, Univ Montpellier, CNRS, INSERM, Montpellier, France

Legends for Supplementary Figures

Suppl. Fig. 1. (A) RT-qPCR of eIF4A3 and Y14 mRNA levels in eIF4A3 and/or Y14 KD in C25Cl48 cells. Data are mean ± SD (n=4) (*$p < 0.05$; multiple Mann-Whitney test). ns, non-significant. **(B)** Representative agarose gels of mis-spliced transcripts analyzed by RT-PCR upon eIF4A3 and/or Y14 KD compared to Ctrl. The percentages of exon exclusion or use of alternative splice sites quantified by the Image Lab software are indicated below the gels.

Suppl. Fig. 2. Electropherograms from QFPCR experiments in eIF4A3 and/or Y14 KD in C25Cl48 cells. Primers used for amplification are indicated above each panel and the identity of each peak is shown (arrows). Full-length QFPCR product (FL).

Suppl. Fig. 3. Sanger sequencing of RT-PCR products in C25Cl48 cells upon eIF4A3 KD. The position of the abnormal junction in the eIF4A3 KD condition is indicated by a dotted line. The sequence shift is visible beyond this position. The primers used for sequencing are indicated on the left side of the sequences.

Suppl. Fig. 4. (A) Comparison of mis-spliced transcript levels determined by DMD-targeted RNA-seq (left panel) and QFPCR (right panel) upon eIF4A3 and Y14 KD in C25Cl48 cells.

Suppl. Fig. 5. (A) Representative agarose gels of out-of-frame (OOF) and in-frame (IF) mis-spliced transcripts analyzed by RT-PCR in UPF1 KD compared to control siRNA (Ctrl) and KD of core EJC components in C25Cl48 cells. The annotations are defined as in Supplementary Fig. 1B. **(B)** Quantification of in-frame (IF) mis-spliced transcripts by QFPCR (n=4) in UPF1 KD compared to Ctrl and eIF4A3 KD. Data are shown as means ± SD (n=4) (*$p < 0.05$; multiple Mann-Whitney test). ns, non-significant. **(C)** Representative electropherograms of QFPCR analysis of PCR fragments amplified with primers 68F-72R or 70F-75R in UPF1 KD compared to Ctrl. **(D)** Quantification of DMD E9, E71 and E78 exclusion level as determined by QFPCR analysis in UPF2 KD compared to UPF1 KD and Ctrl conditions (left panel). Determination by RT-qPCR of the fold change in UPF2 mRNA level in UPF2 KD and EJC components KD (right panel). Data are mean ± SD (n=4) (*$p < 0.05$; multiple Mann-Whitney test). ns, non-significant.

Suppl. Fig. 6. (A) Electropherograms of QFPCR analysis with primers 68F-72R in eIF4A3 KD and Ctrl from HeLa cells compared to the eIF4A3 KD profile in C25Cl48 cells (at bottom). The low level of exon 69 skipping ($E69^-$) is not visible in HeLa cells. **(B)** Representative agarose gels of DMD E78 skipping level (% below the gel) analyzed by RT-PCR upon eIF4A3 KD compared to Ctrl. (∅) non treated HeLa cells.

Suppl. Fig. 7. KD efficiency determined by RT-qPCR of **(A)** eIF4A3 and ASAP/PSAP components and **(B)** MLN51. Data are mean ± SD (n=4) (*$p < 0.05$; multiple Mann-Whitney test). **(C)** Representative agarose gels of mis-spliced transcripts analyzed by RT-PCR under RNPS1 KD for A5.E9 (left panel) and and A5.E70 (right panel) compared to the Ctrl and eIF4A3 KD. The annotations are defined as in Supplementary Fig.1B.

Suppl. Fig. 8. (A) RT-qPCR of Dp427m and Dp71 mRNA level in eIF4A3, Y14, eIF4A3/Y14, UPF1 and UPF2 KD conditions compared to the control (Ctrl) and untreated myoblasts. Data are mean ± SD (n=5) (*p < 0.05, **p < 0.01, multiple Mann-Whitney test). The expression level of Dp71 mRNA is lower and significantly different from that of Dp427m in eIF4A3, Y14, eIF4A3/Y14 KD conditions in C25Cl48 cells differentiated for 3 days (**p < 0.01). The levels of Dp427m and Dp71 are low and not significantly different in untreated myoblasts and in UPF1 KD. In UPF2 KD, Dp71expression is significantly increased compared to the KD control and also compared to Dp427m levels in the same condition.

Supplementary Fig. 1

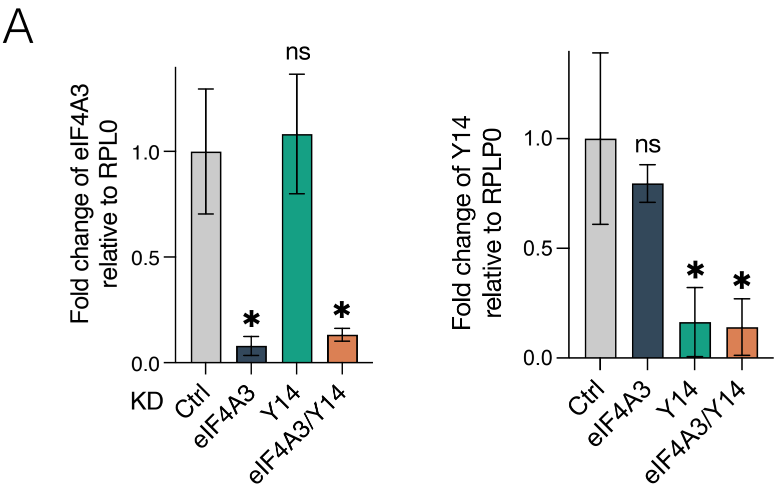

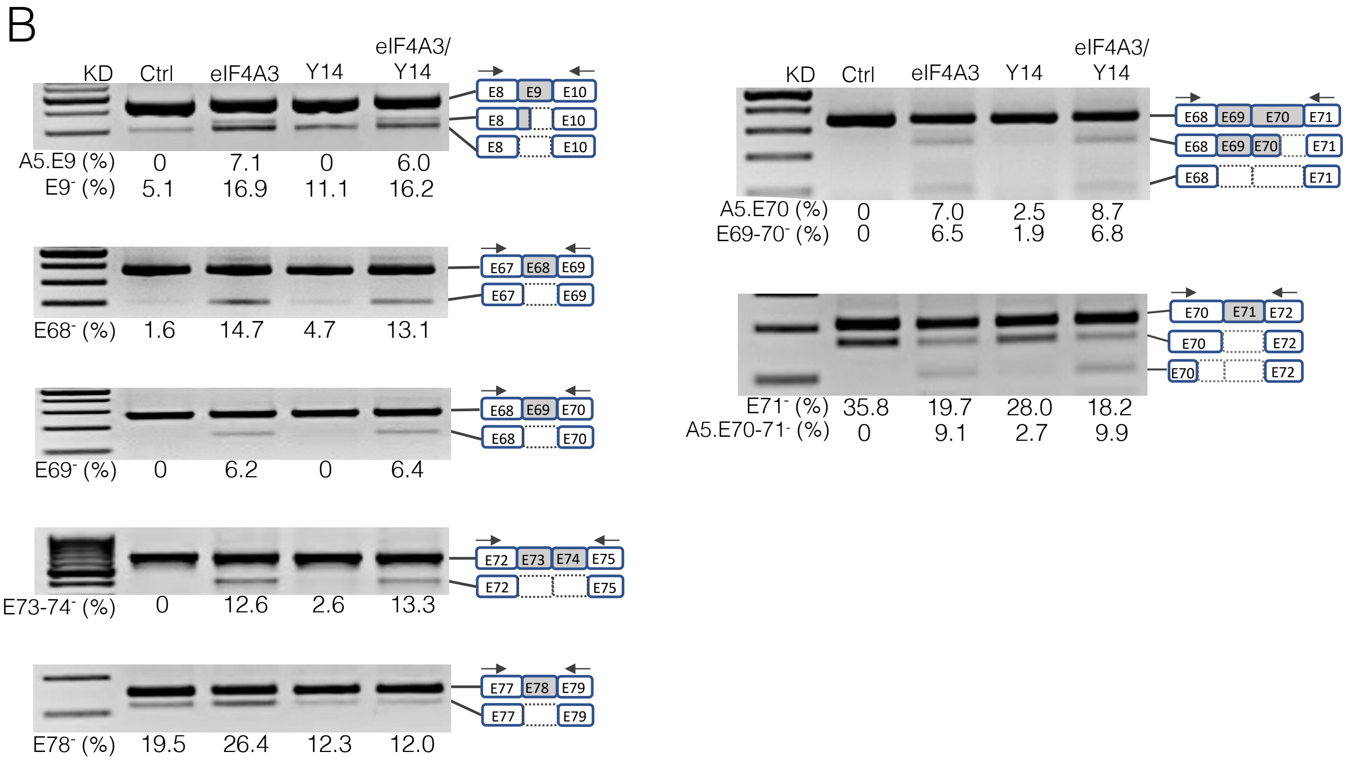

Supplementary Fig. 2

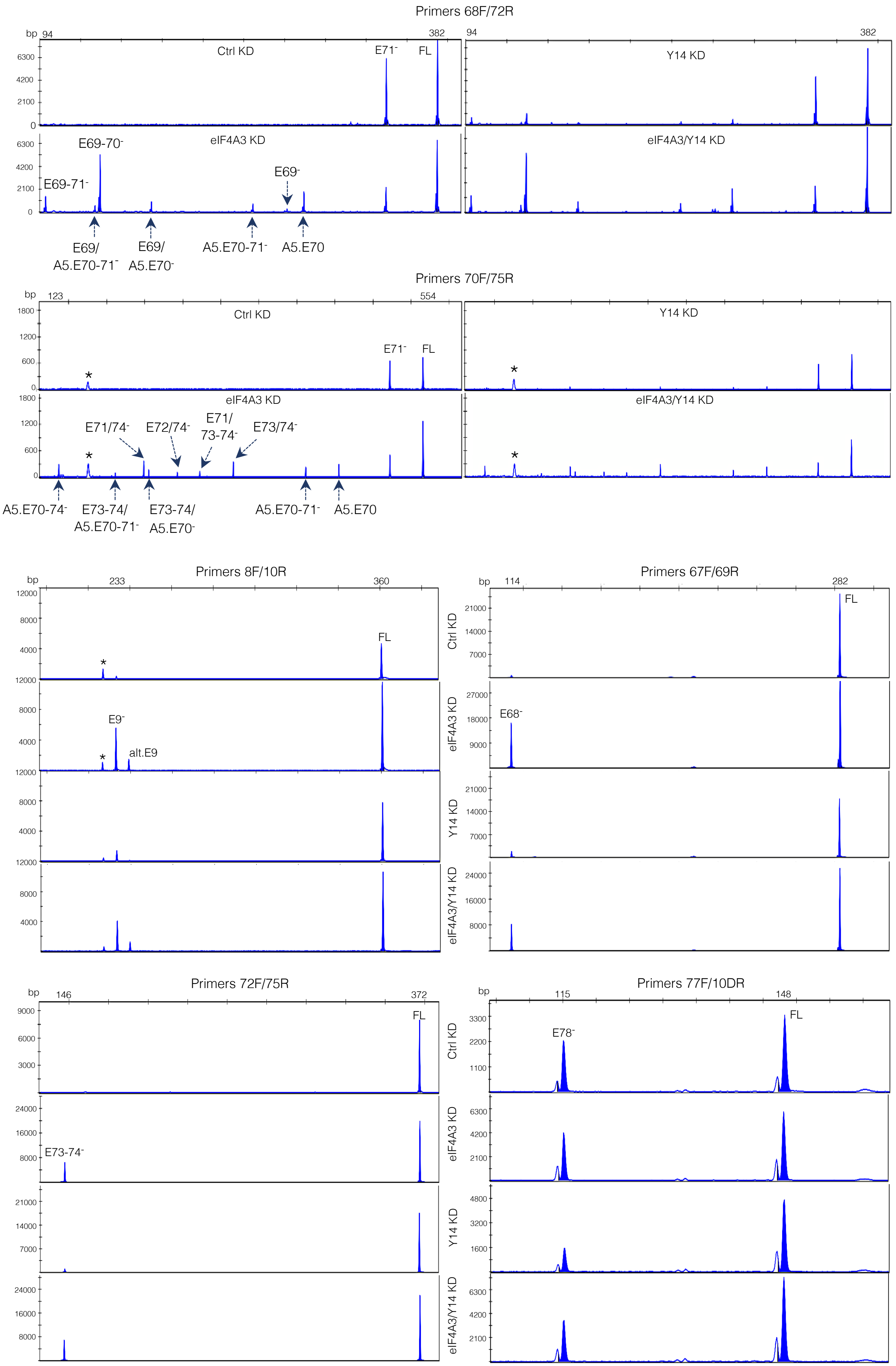

Supplementary Fig. 3

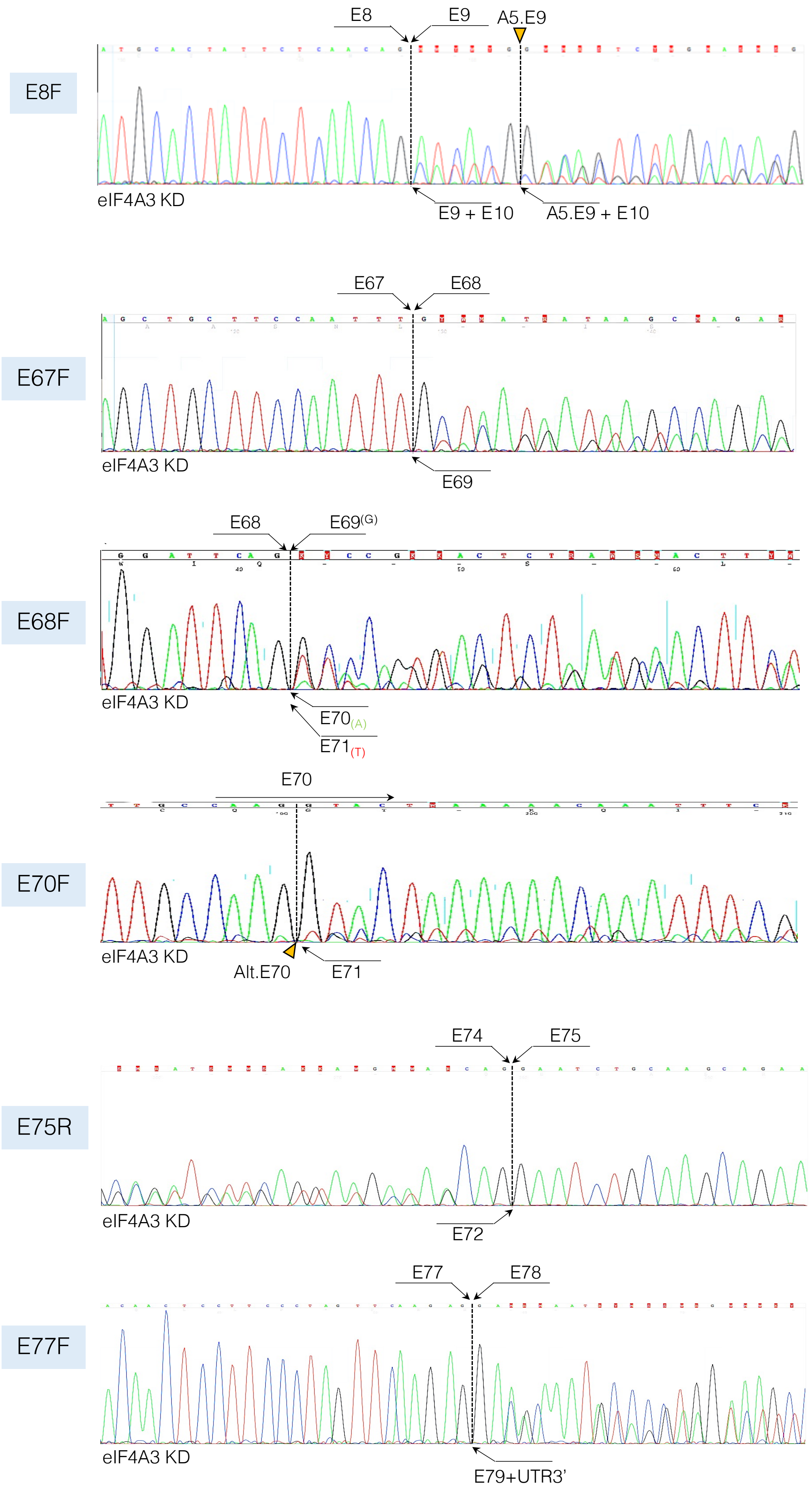

Supplementary Fig.4

A

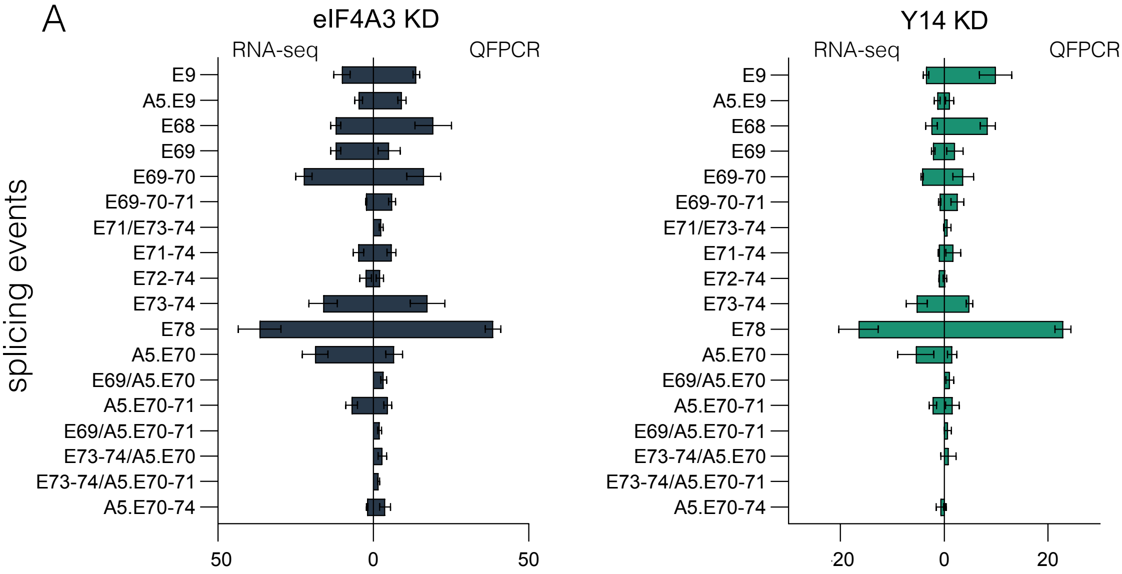

Supplementary Fig. 5

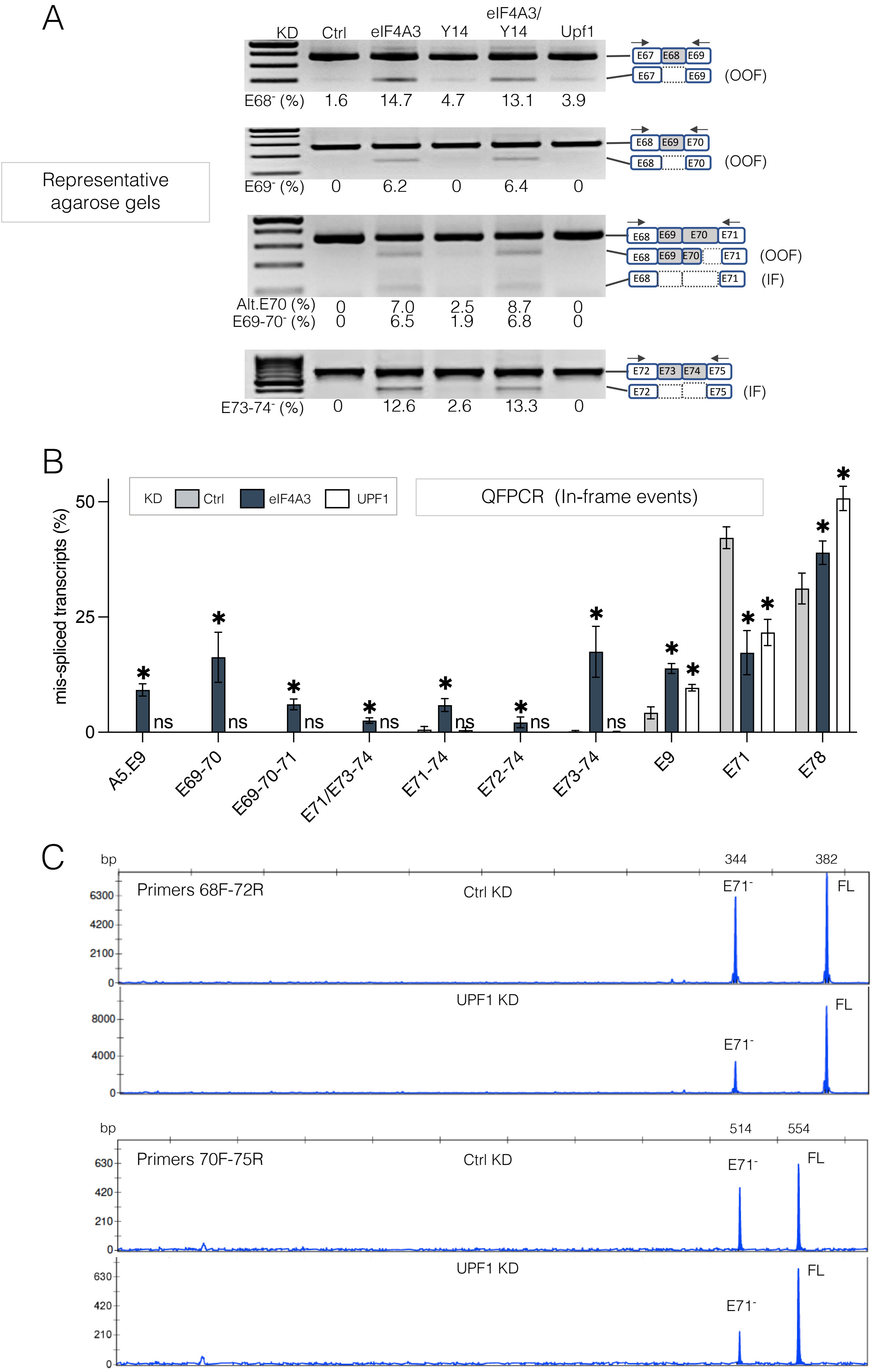

Supplementary Fig. 5

D

Exon 9, Exon 71, Exon 78 skipping
in UPF2 KD (QFPCR) compared to UPF1 KD

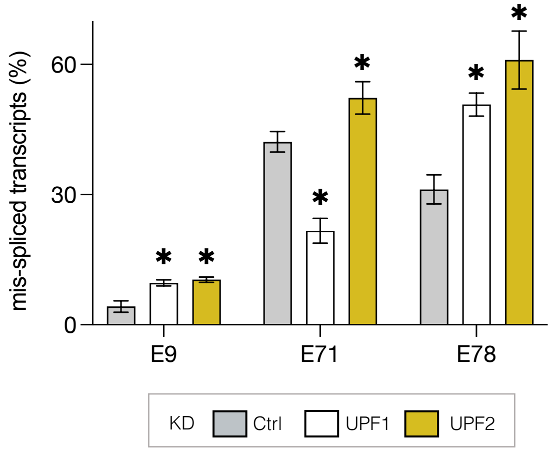

UPF2 (RT-qPCR)
in various KD conditions

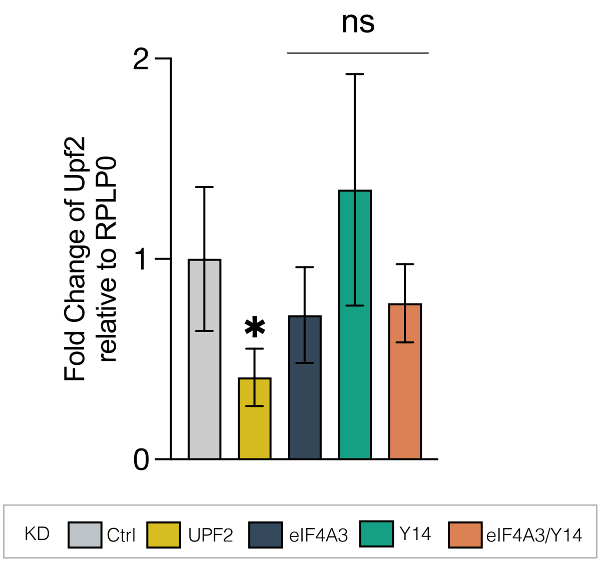

Supplementary Fig. 6

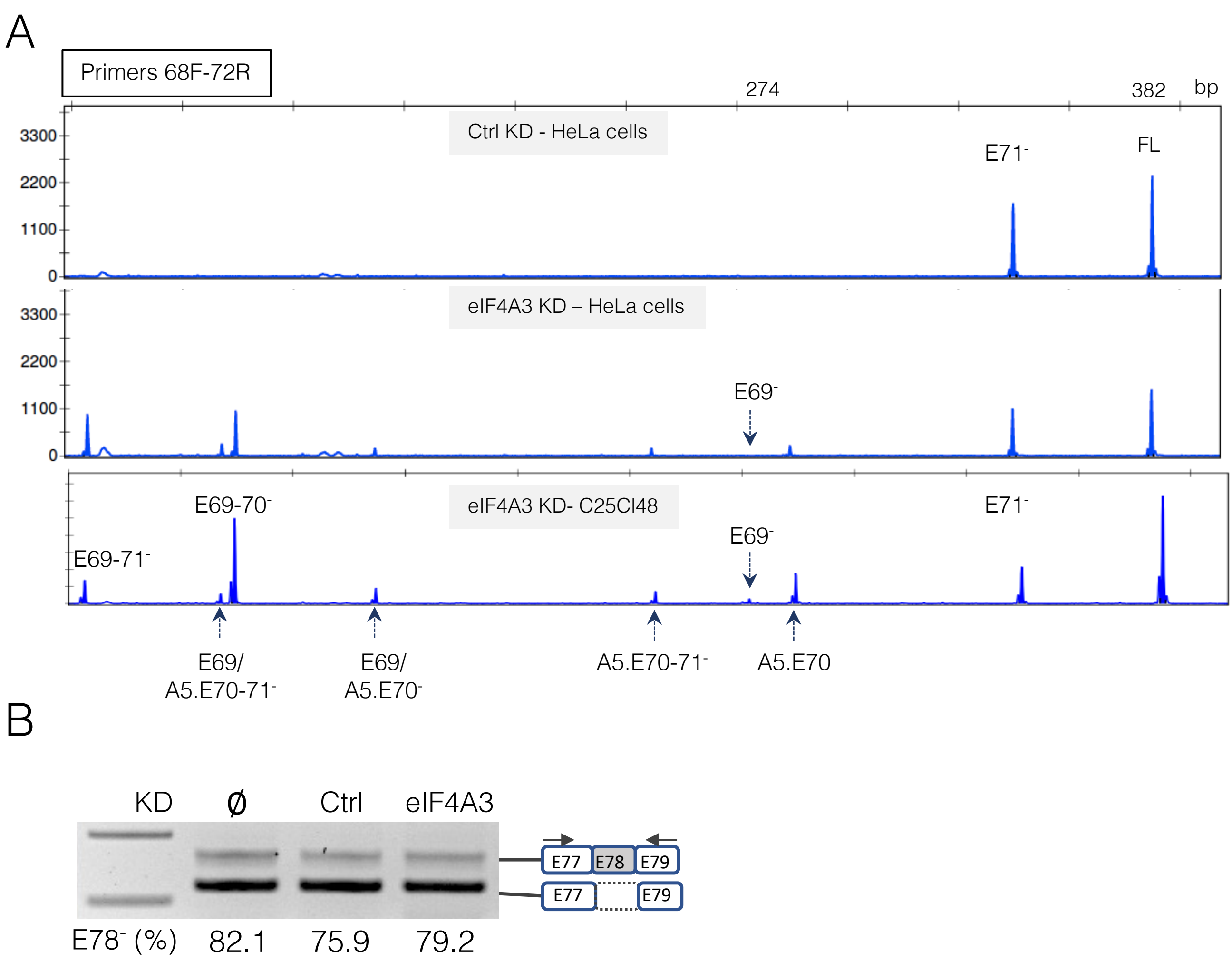

Supplementary Fig. 7

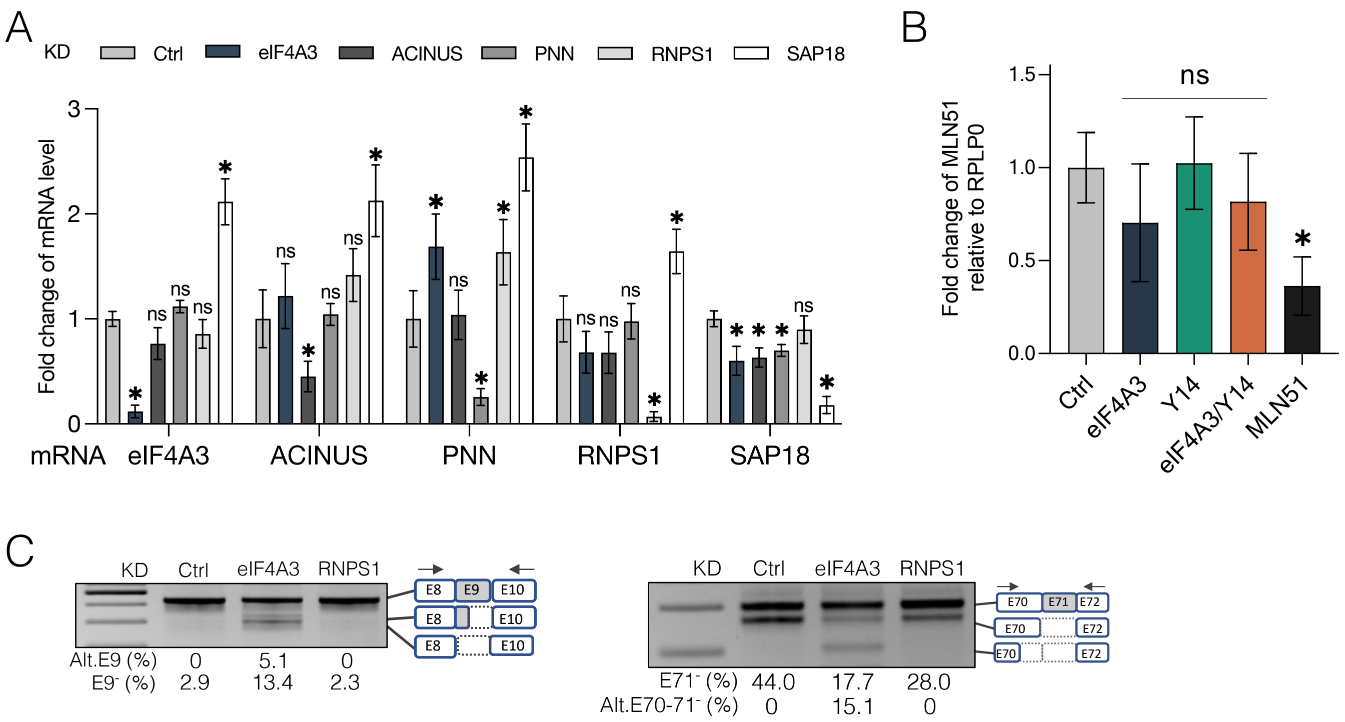

Supplementary Fig.8

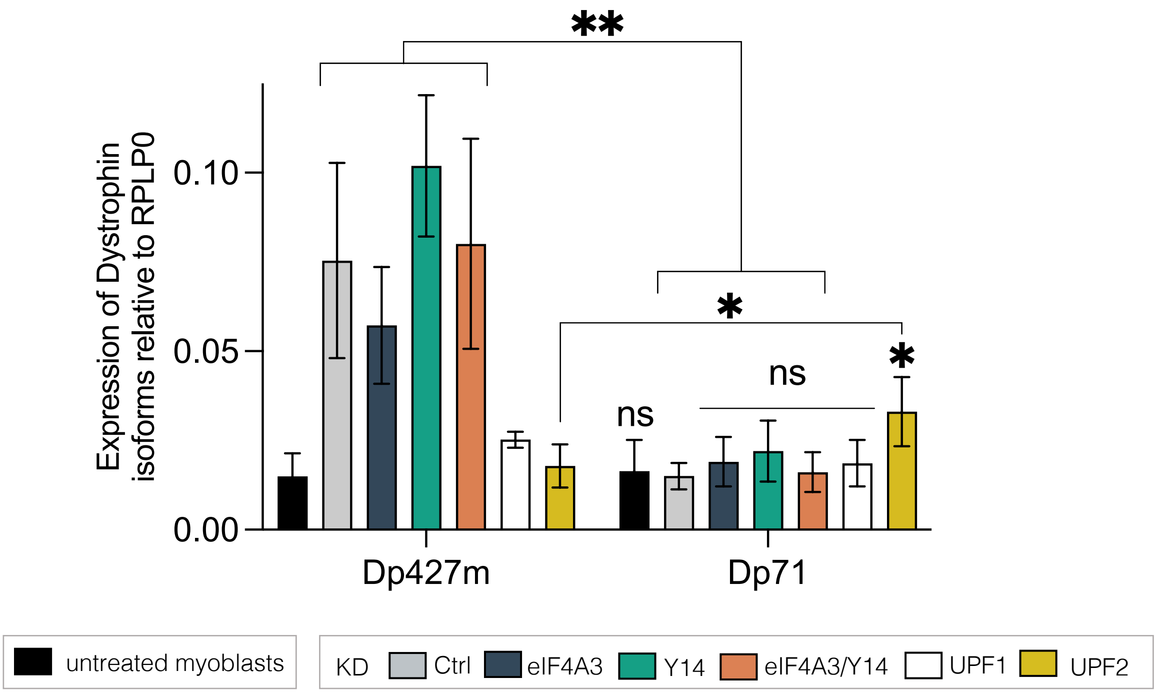

Supplement: Supplementary file 1 — (PDF 1895 KB) [file 18_2024_5188_MOESM1_ESM.pdf]
