# Supplementary material for: The exon junction complex is required for DMD gene splicing fidelity and myogenic differentiation
Source: Cell Mol Life Sci. 2024 Mar 21;81(1):150. doi: 10.1007/s00018-024-05188-1 (PMC10957711; doi:10.1007/s00018-024-05188-1)
Supplement: Supplementary file 3 — (PDF 83 KB) [file 18_2024_5188_MOESM3_ESM.pdf]

siRNA

Names	Sequences	References
siRNA#5 (Ctrl)	UGGUUUACAUGUCGACUAA	Dharmacon (D-001210-05-20)
eIF4A3	AGACAUGACUAAAGUGGAA	Wang et al. (2014)
Y14	CGCUCUGUUGAAGGCUGGA	Wang et al. (2014)
RNPS1	GCAUCCAGCCGCUCAGGAA	Blazquez et al. (2018)
UPF1	GAUGCAGUUCCGCUCCAUU	Wang et al. (2014)
MLN51	CCAGCAUACAUACCUCGGA	Mabin et al. (2018)
UPF2	GGCUUUUGUCCCAGCCAUC	Kim et al. (2005)
ACINUS	GCUCGCUGCCCAAAUCAUU	Wang et al. (2014)
PININ	GGUAAGGUGGCUCAGCGAG	Leu et al. (2012)
SAP18	GGAUGCAACCUUGAAAGAACUGACA	Boehm et al. (2018)

Oligonucleotides sequences used for cloning FLAG-Y14-WT

Names	Sequences (5'->3')
Flag-pcDNA3-NheI-Y14-WT-F	CATCGCTAGCATGGATTACAAGGACGACGACGATAAGGCGGACGTGCTAGATCTTCAC
Flag-pcDNA3-XhoI-Y14-WT-R	TCGACTCGAGTCAGCGACGTCTCCGGTCTGG

Oligonucleotides sequences used for mutagenesis of siRNA-resistant FLAG-Y14-WT

Names	Sequences
siRNA-resistant-FLAG-Y14-F	GGGGTGGATTCTCTTTGTAACTGGAGTCC
siRNA-resistant-FLAG-Y14-R	TCCACCGAGCGTTGTGGTCCGGGTTCATC

RT-PCR oligonucleotides - *DMD* gene *(*,6-FAM-labeled primers used for fluorescent semi-quantitative PCR)*

Names	Sequences (5'->3')
Exon 1F	CTTTCCCCCTACAGGACTCAG
Exon 8F *	AGTGAGCATTGAAGCCATCC
Exon 10R	GCAATGTGTCCTCAGCAGAA
Exon 67F *	CAGTTGGGTGAAGTTGC
Exon 69R	GCCTTTTGCAACTCGACCAGA
Exon 68F *	GCAGAAACTGCCAAGCATCAG
Exon 70R	TCTCGAACATCTTCTCCTGATG
Exon 71R	CAGAAGTTGATCAGAGTAACGG
Exon 70F *	CATCAGGAGAAGATGTTCGAGA
Exon 72R	TCATCGTGTGAAAGCTGAGG
Exon 72F *	CCTCAGCTTTCACACGATGA
Exon 75R	AACCACTCGGAGCAGCATAG
Exon 77F *	AGTCCTCCCCAGGACACAA
3'UTR 10dR	CCAAATCATCTGCCATGTGG

qPCR oligonucleotides

Names	Forward (5'->3')	Reverse (5'->3')
eIF4A3	CCAGCCGAGTGCTTATTTCTAC	CCTGATCTCCCAATTCTGTGTA
Y14	GGACCACAACGCTCTGTTG	CAACTAGAGTATACCCCTTCAG

RNPS1	GGACAAAACCCGAAAGAGGC	CCTCCTGTTGTCGTGTCTGC
UPF1	AGATCACGGCACAGCAGAT	TGGCAGAAGGGTTTTCCTT
UPF2	GAAGATGAGGAAGCCAGCAC	ACATGCAGGGATGCAATGTA
MLN51	AGCCTGACACCAAAAGCACT	TAGGGCCCTTTTTACCCACT
ACINUS	CAGCCACCACACAGAAGAAA	GCGAGAGTCATCAGCATGAA
PININ	ACAAGAATCCACTGTTGCTACTG	CAAAAGCCGCAGTTCTGTCT
SAP18	AATGTACCGTCCAGCGAGTT	GCCCTTCTTTCTAGCTTCTGG
Dp427m	TAGAGGACTGTTATGAAAGAGAAG	CAGGGCATGAACTCTTGTG
Dp71	ATGAGGGAACAGCTCAAAGG	CTGGTCCAAGGCATCACAT
CyclinA	GCACTCTACACAGTCACGG	GTCTCTGGTGGGTTGAGGA
MYF5	CATGCCCGAATGTAACAGTC	CCCAGGTTGCTCTGAGG
MYOD	ACAACGGACGACTTCTATGAC	TGCTCTTCGGGTTTCAGGA
MYOG	ACCCCGCTTCTATGATGG	ACACCGACTTCCTCTTACACA
CKM	CAAGCACCCCAAGTTCG	GTCGTCAATGGACTGGC
ACTA1	GACTTCTCAGGACGACGAATC	CATTTTCTTCCACAGGGCTT
SRSF3	GTGAAAAAAGAAGTAGAAATCGTGG	CTCCTTCTTGGAGATCTGCCGACGAG
PTC-SRSF3	TCCACCTCGTCGCAGAGTCACCATC	TCATGTGAAACGACACCAGCCAAGC
Antibodies		
Names	Dilutions	References
Rabbit-anti-eIF4A3	1:20000	Gift from Hervé Le Hir Lab
Rabbit-anti-Y14	1:250	Gift from Hervé Le Hir Lab
Rabbit-anti-MLN51	1:500	Gift from Hervé Le Hir Lab
Goat-anti-UPF1	1:500	Bethyl (#A300-038A, RRID:AB_2288326)
Rabbit-anti-RNPS1	1:1000	Proteintech (#10555-1-AP, RRID:AB_2181682)
Rabbit-anti-ACINUS	1:100	Thermo Fisher Scientific (#720313, RRID:AB_2665122)
Rabbit-anti-PININ	1:250	Proteintech (18266-1-AP, RRID:AB_10642138)
Mouse-anti-SAP18	1:250	Santa Cruz (#sc-365377, RRID:AB_10851473)
Mouse-anti-Tubulin	1:20000	Proteintech (#66031-1-Ig, RRID:AB_11042766)
Rabbit-anti-GAPDH	1:4000	Santa Cruz (#sc-25778, RRID:AB_10167668)
Mouse-anti-MyHC	1:8000	Sigma (#M1570, RRID:AB_2147168)
Mouse-anti-TroponinT	1:2500	Sigma (T6277, RRID:AB_261723)
Mouse-anti-Dystrophin	1:100	GeneTex (GTX01871)
Cell lines		
C25Cl48		KM155C25, RRID:CVCL_6B24
HeLa cells		RRID:CVCL_0030

Supplementary Table 1.
Tables of oligonucleotide sequences and antibodies used in this study.
