# Supplementary material for: The exon junction complex is required for DMD gene splicing fidelity and myogenic differentiation
Source: Cell Mol Life Sci. 2024 Mar 21;81(1):150. doi: 10.1007/s00018-024-05188-1 (PMC10957711; doi:10.1007/s00018-024-05188-1)
Supplement: Supplementary file 4 — (PDF 100 KB) [file 18_2024_5188_MOESM4_ESM.pdf]

		siRNA in C25cl48 cell line						ΔSJ			
		Ctrl KD		eIF4A3 KD		Y14 KD		eIF4A3 KD vs Ctrl KD		Y14 KD vs Ctrl KD	
Splicing events	Reading Frame	RNA_seq n=4	RT_QFPCR n=4	RNA_seq n=2	RT_QFPCR n=4	RNA_seq n=2	RT_QFPCR n=4	RNA_seq	RT_QFPCR	RNA_seq	RT_QFPCR
E9	In Frame	0,75	4,22	10,08	13,84	3,46	9,90	9,33	9,62	2,71	5,68
A5.E9	In Frame	0,00	0,00	4,73	9,19	1,35	1,05	4,73	9,19	1,35	1,05
E68	Out of Frame	0,32	3,71	12,05	19,31	2,43	8,38	11,73	15,60	2,11	4,67
E69	Out of Frame	0,00	0,00	12,06	5,11	2,12	2,07	12,06	5,11	2,12	2,07
E69-70	In Frame	0,00	0,00	22,35	16,26	4,24	3,67	22,35	16,26	4,24	3,67
E69-70-71	In Frame	0,00	0,00	2,27	6,02	0,87	2,55	2,27	6,02	0,87	2,55
E71/E73-74	In Frame	0,00	0,06	0,00	2,54	0,00	0,60	0,00	2,48	0,00	0,54
E71-74	In Frame	0,64	0,59	4,77	5,90	1,05	1,77	4,13	5,31	0,41	1,18
E72-74	In Frame	0,00	0,00	2,49	2,15	0,99	0,15	2,49	2,15	0,99	0,15
E73-74	In Frame	0,00	0,18	16,09	17,47	5,29	4,86	16,09	17,29	5,29	4,68
E78	Terminal stop codon shift	29,30	31,59	36,57	38,58	16,49	22,89	7,27	6,99	-12,81	-8,70
A5.E70	Out of Frame	0,00	0,00	18,69	6,71	5,47	1,55	18,69	6,71	5,47	1,55
E69/A5.E70	Out of Frame	0,00	0,00	0,00	3,33	0,00	1,10	0,00	3,33	0,00	1,10
A5.E70-71	Out of Frame	0,00	0,00	6,97	4,68	2,20	1,58	6,97	4,68	2,20	1,58
E69/A5.E70-71	Out of Frame	0,00	0,00	0,00	2,05	0,00	0,74	0,00	2,05	0,00	0,74
E73-74/A5.E70	Out of Frame	0,00	0,00	0,00	2,90	0,00	0,85	0,00	2,90	0,00	0,85
E73-74/A5.E70-71	Out of Frame	0,00	0,00	0,00	1,74	0,00	0,00	0,00	1,74	0,00	0,00
A5.E70-74	Out of Frame	0,00	0,08	1,91	3,80	0,70	0,13	1,91	3,72	0,70	0,05

Supplementary Table 3.
Comparison between the levels of mis-spliced transcripts detected by *DMD*-targeted RNA-Seq and QFPCR in Ctrl, eIF4A3 and Y14 KD. The ΔSJ shows the changes in the splicing event between EJC KD and the si-Ctrl condition.
